# Effect of Ionizing Radiation on the Chemical Structure and the Physical Properties of Polycaprolactones of Different Molecular Weight

**DOI:** 10.3390/polym10040397

**Published:** 2018-04-03

**Authors:** Rodrigo Navarro, Guillermina Burillo, Esbaide Adem, Angel Marcos-Fernández

**Affiliations:** 1 Instituto de Ciencia y Tecnología de Polímeros (CSIC), Juan de la Cierva 3, 28006 Madrid, Spain; rnavarro@ictp.csic.es; 2 Instituto de Ciencias Nucleares, Universidad Nacional Autónoma de México, Circuito Exterior, Ciudad Universitaria, 04510 Ciudad de México, Mexico; burillo@nucleares.unam.mx; 3 Instituto de Física, Universidad Nacional Autónoma de México, Circuito Exterior, Ciudad Universitaria, 04510 Ciudad de México, Mexico; esbaide@fisica.unam.mx

**Keywords:** polycaprolactone, electron beam irradiation, gamma rays, mechanical properties, degradation mechanism

## Abstract

Polymers used in the biomedical sector can be exposed to ionizing radiation (X-ray, gamma) in vivo as implants or ex vivo for sterilization purposes (gamma, electron beam). This ionizing radiation can, at certain levels, cause degradation of the polymer. Polycaprolactones (PCL) of different molecular weights were irradiated with electron beam and the changes in their chemical structure and physical properties with the dose were evaluated. Electron beam irradiation produced crosslinking and chain scission in the PCL chain without significant predominance of one mechanism over the other. Minimum dose for gelation decreased with the increase in PCL molecular weight whereas crosslinking efficiency was almost independent of PCL molecular weight. Carboxylic groups, hydroxyl groups and new saturated hydrocarbon species were detected by proton nuclear magnetic resonance (NMR). These species were consistent with a mechanism where chain scission could take place at any bond in the PCL chain with preference in the –COO–CH_2_– bond. Crosslinking decreased significantly the crystallization temperature of PCL. Tensile properties decreased continuously with the increase in dose. Irradiation with gamma rays produced a faster decay in mechanical properties than electron beam.

## 1. Introduction

Polycaprolactone (PCL) is a hydrophobic, semicrystalline, non-toxic, biodegradable polymer with good mechanical properties. Its good solubility in common solvents, low melting point (59–64 °C), Food and Drug Administration (FDA) approval and outstanding blend compatibility has led to an extensive research into its potential application in the biomedical field. PCL is very easy to process and manipulate into a large range of shapes and sizes and it has been successfully incorporated as an implantable biomaterial in several applications such as sutures and wound dressings, cardiovascular tissue engineering, nerve regeneration, bone tissue engineering and controlled delivery devices [1,2].

In order to use these biomedical implants in practice, it will be necessary to sterilize them by methods that are conventionally used for other implants. Steam sterilization involves subjecting the product to steam at 121 °C which is impractical for PCL because the polymer would soften in these conditions leading to deformation of the matrix form. The same problem of matrix dimensional stability is found when heat sterilization is used. When high temperature is a problem as for PCL, the use of a gas such as ethylene oxide at ambient temperature is an option. However, ethylene oxide is known to soften and plasticize PCL and the residual vapours left in the device were found to be mutagenic, carcinogenic and allergic. Thus, ionizing radiation (electron beam or gamma rays) is likely to be the method of choice for PCL implants [1,3].

Ionizing radiation is highly penetrative and kills bacteria by breaking down bacterial DNA, thereby inhibiting bacterial division. Energy of electron beam or gamma rays passes through the material, disrupting the pathogens that cause contamination. Such photon-induced damage at the molecular level leads to the death of microorganisms [4]. In addition to killing bacterial cells, radiation may also affect physical and chemical properties of the polymers. The ionizing radiation excites and ionizes atoms and polymer molecules on the targeted substrate and the radicals produced, promote the decomposition and/or crosslinking of the polymer as well as implantation of new functional groups, which depends on the surrounding media during the treatment [5].

A minimum dose of 25 kGy was routinely applied for the sterilization of many medical devices, pharmaceutical products and biological tissues. Presently, as recommended by the International Organization for Standardization (ISO), the sterilization dose must be set for each type of product depending on its characteristics and the load of microbes [4]. It is important to optimize the dosage of ionizing radiation required to successfully sterilize the PCL devices as well as to study the physicochemical changes induced in the polymeric matrix. Some studies on the effect of gamma rays [3,4,6,7,8,9,10,11] or electron beam [11,12,13,14,15,16,17,18] on the structure and properties of PCL can be found in literature. Part of these studies were performed at sterilization doses up to 75 kGy [3,4,9,11] and some of them reached higher doses [6,7,8,10,12,13,14,15,16,17,18]. All these studies were carried out with a single PCL material with a defined molecular weight [3,4,6,7,8,9,11,12,13,14,15,16,17,18] except one, where the effect of gamma rays on the gelation dose and the thermal properties of three PCLs of different molecular weight was evaluated [10].

The important properties for a bioresorbable scaffold are its rate of degradation, its mechanical strength and its ability to support cell growth. Irradiation with gamma rays at a sterilizing dose of 30.8 kGy significantly decreased the rate of degradation of a PCL with molecular weight not given and did not affect the chondrocyte attachment and growth. For the mechanical properties of this PCL, yield stress increased significantly with irradiation but not the stress at break [3]. Mechanical strength is important for any application where the tissue has a mechanical role in the body (as is the case for most connective tissues, e.g., cartilage) or is subjected to appreciable mechanical loads. For PCL, the effect of dose on the mechanical properties has been studied in single samples and there are not studies on the effect of ionizing radiation on the mechanical properties of PCL with different initial molecular weight. When PCL was irradiated with electron beam or gamma rays, strain at break decreased respect to the non-irradiated material in all cases reported [6,7,15] except for one [3]. Tensile stress at break generally increased at low irradiation doses [3,17] but when high doses were applied, stress at break passed through a maximum at approximately 160–200 kGy dose and then decreased continuously at higher doses [7,15]. Thus, results for the effect of ionizing radiation on the mechanical properties of PCL are somehow ambiguous and a general trend has not been derived. In the only study where PCL of different molecular weight was irradiated with gamma rays, mechanical properties were not measured [10] and therefore, to our knowledge, there is not study on the influence of the molecular weight of the PCL on the effect of the ionizing radiation on its mechanical properties.

In this work, we report the effect of electron beam irradiation on the chemical structure and the physical properties of several PCLs of different molecular weight with a special focus on the effect on the mechanical properties. In addition, we compare the differences in the effect on the mechanical properties when PCL was irradiated with electron beam or with gamma rays.

## 2. Materials and Methods

### 2.1. Materials and Characterization Techniques

Polycaprolactones of molecular weights of 25,000, 37,000, 43,000, 50,000 and 80,000 g·mol^−1^, as given by the supplier, with commercial names CAPA™ 6250, 6400, 6430, 6500 and 6800 respectively, were provided by Perstorp (Cheshire, UK).

The thermal transitions of the samples were analysed by Differential Scanning Calorimetry (DSC) on a Mettler Toledo DSC 822e calorimeter (Schwerzenbach, Switzerland) equipped with a liquid nitrogen accessory. Discs cut from sheets weighing approximately 30–40 mg were sealed in aluminium pans. Samples were heated, from 25 to 90 °C at a rate of 10 °C·min^−1^, cooled to −90 °C at a rate of −10 °C·min^−1^, maintained for 5 min at this temperature and re-heated from −90 to 90 °C at a rate of 10 °C·min^−1^. All scans were carried out under a constant nitrogen purge. Melting points (*M*_p_) and crystallization temperatures (*T*_c_) were taken as the maximum of the endothermic transition and the minimum of the exothermic transition respectively, whereas glass transition temperatures (*T*_g_) were taken as the midpoint of the change in heat capacity. The crystallinity of the samples was calculated by taking the enthalpy of fusion of the 100% crystalline polymer as 16.9 kJ·mol^−1^ (148.2 J·g^−1^) [19].

Tensile properties were measured at ambient temperature in a MTS Synergie 200 testing machine (Eden Prairie, MN, USA) equipped with a 100 N load cell. Type 4 dumbbell test pieces (according to ISO 37) were cut from the moulded sheets. A cross-head speed of 5 mm·min^−1^ was used. Strain was measured from cross-head separation and referred to 10 mm initial length. A minimum of 5 samples were tested for each material.

Molecular weights were determined by size exclusion chromatography (SEC), using a Waters (Milford, MA, USA) 1515 gel permeation chromatograph equipped with a refractive index detector Waters IR2414. A set of HR4, HR1 and HR0.5 Waters columns conditioned at 35 °C was used to elute samples at 1 mL·min^−1^ flow rate with HPLC-grade THF as solvent. Polystyrene standards (2970 to 200,000 g·mol^−1^) were used for the calibration. Standards above 200,000 g·mol^−1^ showed that above this value the limit of total exclusion of the columns was reached.

Solution nuclear magnetic resonance (NMR) spectra were recorded at room temperature in a Varian Unity Plus 400 instrument (Palo Alto, CA, USA) using deuterated chloroform (CDCl_3_) as solvent. Spectra were referenced to the residual solvent signal at 7.26 ppm.

### 2.2. Preparation of PCL Sheets

Sheets from the PCLs were prepared by melting in a 10 cm × 10 cm × 1 mm steel mould with the following procedure. PCL pellets (approximately 14 g) were charged in the open mould in a vacuum oven at 120–180 °C (120 °C for CAPA™ 6250, 140 °C for CAPA™ 6400 and 6430, 160 °C for CAPA™ 6500 and 180 °C for CAPA™ 6800) and high vacuum applied. After degasification for 2 h at this temperature, the vacuum was released, the mould closed and let to cool at room temperature overnight. Next day the PCL sheet, free from defects, was de-moulded.

### 2.3. Irradiation of the Samples

Rectangles of approximately 3 cm × 1 cm for gel content measurements and type 4 dumbbell test pieces (according to ISO 37) for thermal and mechanical properties were cut from the PCL sheets. The samples were introduced into 9 cm × 6 cm polyethylene bags and sealed in air. Part of the samples were irradiated with an electron-beam accelerator, using a Van de Graaff source, 1.3 MeV energy and a beam current of 5 µA at a dose rate of 4.8 kGy·min^−1^ and doses from 10 to 1000 kGy in air at room temperature. And part of the samples was irradiated with a ^60^Co-source (Gammabeam 651-PT, Nordion International Inc., Ottawa, ON, Canada) with an activity of 63,000 Ci at a dose rate of 9.87 kGy·h^−1^ and doses from 25 to 200 kGy in air at room temperature. Determination of dose rate for gamma ray source was carried out with modified Fricke (ferrous sulphate dosimeter modified by addition of cupric sulphate) [20]. Determination of dose rate for electron beam was done using 1 cm × 1 cm radiochromic dosimeters series FWT-60-00 from Far West Technology, Inc. (Goleta, CA, USA).

### 2.4. Gel Content Determination

To evaluate the gel content, 150–200 mg of e-beam irradiated PCLs were immersed in 10 mL chloroform for 2 h at room temperature. Solvent was decanted and 10 mL of fresh chloroform were added. After 24 h at room temperature, decantation and addition of fresh solvent was repeated and left for further 24 h. Finally, chloroform was decanted and the resulting gel was dried in air for 2 h and in vacuum at room temperature overnight. The remaining dry solid was weighed. The gel content was calculated from the weight ratio of the insoluble fraction after irradiation and the sample prior to irradiation. The decanted chloroform extracts were evaporated and the molecular weight of the soluble fraction of the irradiated samples measured by size exclusion chromatography (SEC).

## 3. Results and Discussion

### 3.1. Polycaprolactones Characterization

Before irradiation, the materials were characterized for its molecular weight and physical properties.

In Figure S1 in the Supporting Information file it is depicted the molecular weight given by the supplier against the molecular weight in the peak maximum obtained by SEC. As it can be seen, the points fitted a straight line except for the polycaprolactone of 37,000 g·mol^−1^ molecular weight, that lied significantly outside the line. If all the data were fitted except for the PCL of 37,000 g·mol^−1^, the correlation factor was good (0.998) and the difference between the experimental data and the calculated values with the straight line equation was below 7%. Using the equation, the new molecular weight calculated for the PCL of 37,000 g·mol^−1^ was lower, 32,000 g·mol^−1^. This calculated value was taken for the rest of the study. Polydispersity of the samples was similar in between 1.49 and 1.51 except for the PCL of 80,000 g·mol^−1^ that had a value slightly higher of 1.60.

Representative tensile stress-strain curves and tensile stress and strain results for the polycaprolactones are represented in Figures S2 and S3 in the Supporting Information file respectively. As it can be seen in Figure S2, the PCL with the lowest molecular weight was fragile whereas the rest of the PCLs were tough, with a yielding point at approximately 20% strain. For the PCL with the highest molecular weight, the tensile specimens became very thin during the test and always escaped from the grip before rupture thus the values in Figure S3 are the values recorded until the specimens escaped and the real value for tensile stress and strain will be higher. As expected, tensile stress and strain increased almost linearly with the increase in molecular weight until a molecular weight of 50,000 g·mol^−1^ and it is awaited to increase further at a molecular weight of 80,000 g·mol^−1^.

In semi-crystalline materials, the mechanical properties are strongly affected by the fraction of crystallinity. To evaluate if the variation in the mechanical properties with the molecular weight was influenced by the crystallinity, the thermal properties of the PCLs were evaluated by DSC. In Figure S4 in the Supporting Information file the crystallinity of the PCLs pristine pellets and after 30 min and 10 days recrystallization at ambient temperature from the melt is represented. Crystallinity in the initial pellets was high and similar for all the PCLs (around 58%) except for PCL of 80,000 g·mol^−1^ that showed a slightly lower value. After recrystallization from the melt, crystallinity decreased with the increase in molecular due to the increased restriction to ordering when chain length increased, as observed by other authors [21]. At longer times, it is expected that crystallinity could reach the values shown by the initial pellets, that is, crystallinity will be similar for all the PCLs. Therefore, the crystallinity is not responsible for the increase in the mechanical properties with the increase in PCL molecular weight and it is the proper increase in the molecular weight of the PCL the main factor influencing the increase in mechanical properties. When PCL molecular weight increases, the entanglement density of the amorphous phase and the interconnectivity of amorphous phase and crystals increase, both effects leading to the increase in mechanical properties [21].

### 3.2. Gel Content of Irradiated Samples

**Figure 1:**
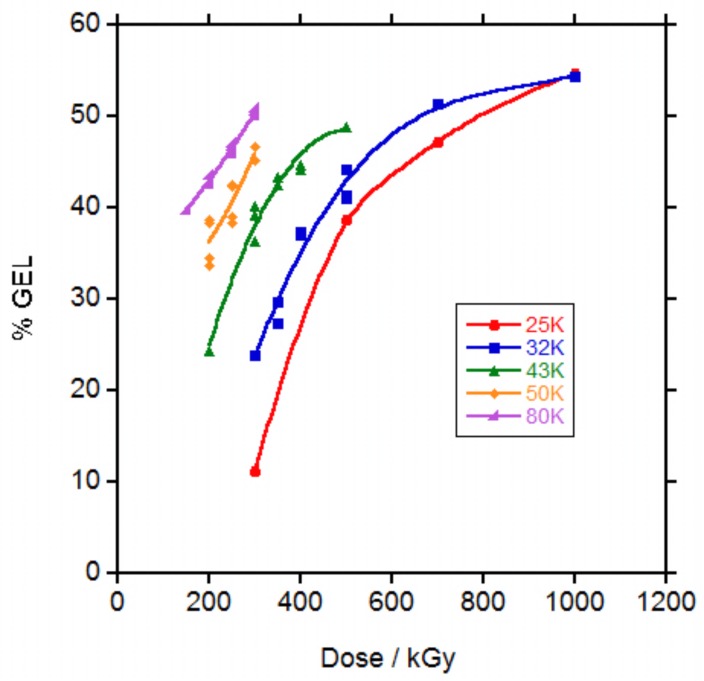
Gel percentage in the irradiated polycaprolactones (PCLs) vs dose.

It is well known that irradiation with electrons and more generally with ionizing radiation, produces simultaneously chains crosslinking and chain scission [22]. In order to evaluate the crosslinking of the polycaprolactones, samples were immersed in chloroform to extract the soluble chains and the percentage of the remaining crosslinked material calculated. In Figure 1, the gel percentage versus irradiation dose is represented.

The percentage of gel at a certain dose was highly dependent on the molecular weight of the polycaprolactone. At 50 kGy dose, none PCL showed evidence of gel. At 100 kGy dose, only PCL 80,000 (80 K) showed gel and at 150 kGy, PCL 50,000 (50 K) and PCL 43,000 (43 K) presented gel. For PCL 32,000 (32 K) and PCL 25,000 (25 K), 200 and 300 kGy were necessary to obtain gel respectively. For some samples at low doses, the gel content could not be measured because the gel could not be separated and only the doses for which the gel content could be measured are represented in Figure 1. For the same dose, the higher the molecular weight of the PCL the higher the gel content. For the PCLs of lower molecular weight, it seems that at higher doses the gel content tended to a plateau.

It has already been shown that when PCL was irradiated with ionizing radiation (electron beam or gamma rays) in vacuum [6,8], inert atmosphere [12,13] or in the melted state in supercooled conditions [8], gel appeared at a lower dose that when irradiation was carried out in the presence of air at ambient temperature. In addition, comparison with results found in literature is difficult because in some cases the molecular weight given is the molecular weight given by the supplier and in other cases is the molecular weight measured by SEC and the difference is very big in between both data al shown in Figure S1 in the Supporting Information file. Gel content given for a PCL 80 K weight given by the supplier (coincident with the PCL 80 K of this study based on the coincidence in the melt flow index data given by the supplier) irradiated with electron beam in air was 22% at 150 and 200 kGy doses [17] which is lower than the values of approximately 40% and 43% respectively found in this study. When other authors applied a 270 kGy dose with an electron beam in air at the same PCL 80 K a 55% gel content was found [13] which is slightly higher than the 45% and 50% values found for 250 and 300 kGy dose respectively in this study. In both cases gel appeared at doses above 50 and 70 kGy respectively, similar to the value in between 50 and 100 kGy necessary for gel formation in the PCL 80 K of this study.

**Figure 2:**
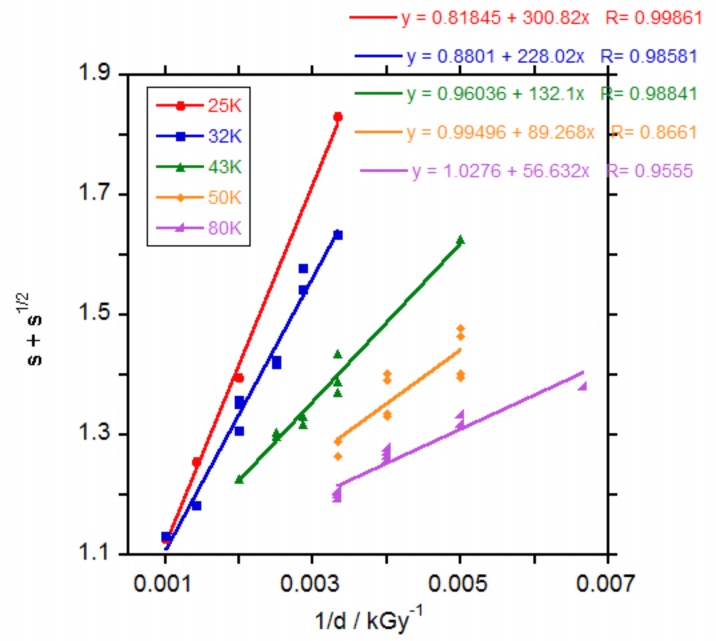
Charlesby-Pinner plot of irradiated PCLs.

The random radiation crosslinking of thermoplastic polymers is described by the classical Charlesby-Pinner equation:

$$s+s^{1/2}=\;\frac{p_{o}}{q_{o}}+\;\frac{1}{q_{o}\;\mu_{1}\;d}$$

where *s* is sol fraction, *p*_o_ is degradation density, *q*_o_ is crosslinking density, *μ*_1_ is initial molecular weight (*M*_n_) and *d* is radiation dose. Irradiation with electrons causes both random chain scission and random interchain bond formation (crosslinking) and the ratio of scission to cross-linking, *p*_o_*/q*_o_, represents the inverse crosslinking efficiency of a polymer system at a specific dose. In a classical Charlesby-Pinner analysis plot, *s + s*^1/2^ is plotted against *1/d* and a linear fit of the data yields a positively sloping trend line with intercepts at *s + s*^1/2^ equals 2 and *1/d* equals 0. The *s + s*^1/2^ equals two intercept represents *d*_o_, the minimum dose to gelation and the *1/d* equals 0 intercept represents the inverse crosslinking efficiency, represented by *p*_o_*/q*_o_ [23]. The Chalesby-Pinner plot of the results for the irradiated PCLs, Figure 2, produced fairly linear fits with the slope of the fit decreasing with the increase in the molecular weight of the PCL, related to a decrease in crosslinking efficiency (*q*_o_*/p*_o_).

The calculated minimum dose for gelation (*d*_o_) increased with the decrease in the molecular weight of the PCL as observed in the measurements of gel percentage. In Figures S5 and S6 in the Supporting Information file the values for *q*_o_*/p*_o_ and *d*_o_ versus PCL molecular weight respectively are represented. Calculated values for *d*_0_ (255, 204, 127, 89 and 58 kGy for PCL 25, 32, 43, 50 and 80 K respectively, Figure S6) agreed with the observed values for the apparition of gel. It was expected that the longer the PCL chains, the lower the number of crosslinks necessary to produce gel, as found by other researchers for PCLs of 40, 50 and 70 K molecular weight as given by the supplier irradiated with gamma rays in air [10]. The values found for *d_o_* were 182, 91 and 41 kGy respectively which are close to the values found in this study for PCL 32, 50 and 80 K respectively.

The calculated crosslinking efficiency (*q_o_/p_o_*) decreased from 1.22 to 0.97 when PCL molecular weight increased (Figure S5 in the Supporting Information file). This value, close to 1, demonstrates that chain scission and crosslinking have similar contributions without significant predominance of one mechanism over the other. A similar value close to 1 has been found by other authors for PCL irradiated with gamma rays [6,7] and only in one work values below 1 (0.6–0.8) were calculated for PCLs of different molecular weight irradiated with gamma rays [10]. In that work, it was also found that crosslinking efficiency increased with the increase in PCL molecular weight, contrary to our results. For the electron beam irradiation of PCL 80 K weight as giver by the supplier in air or Argon at 20 or 60 °C, when Charlesby-Pinner analysis was performed, the authors found that data did not fit a straight line but bent. They argued that it was necessary an initial molecular weight distribution (*M*_w_/*M*_n_) equal to 2 to obtain a straight line. Initial molecular weight distribution for our PCLs was 1.55–1.62 as measured by SEC. Probably due to the limited number of doses in our work the curvature of the data could not be clearly seen and the trend using Charlesby-Pinner analysis produced erroneously the unexpected decay in crosslinking efficiency with the increase in PCL molecular weight seen in Figure S5. Nevertheless, the crosslinking efficiency values for all the PCLs are around 1 showing the similar contribution of chain scission and crosslinking when PCLs are irradiated with an electron beam. This effect is very different from the effect of peroxides in PCLs for which crosslinking predominates strongly over chain scission, with values for crosslink efficiency of 6.7 or above [24,25].

### 3.3. Molecular Weight of Irradiated PCLs

Molecular weight was measured by SEC for the irradiated PCLs before gelation and for the soluble fraction of the crosslinked PCLs after gelation. In the particular case of PCL 25 K, due to its low mechanical properties, it was excluded from further studies. 

In Figures S7–S10 in the Supporting Information file, the SEC curves for the non-irradiated and irradiated PCLs are presented.

**Figure 3:**
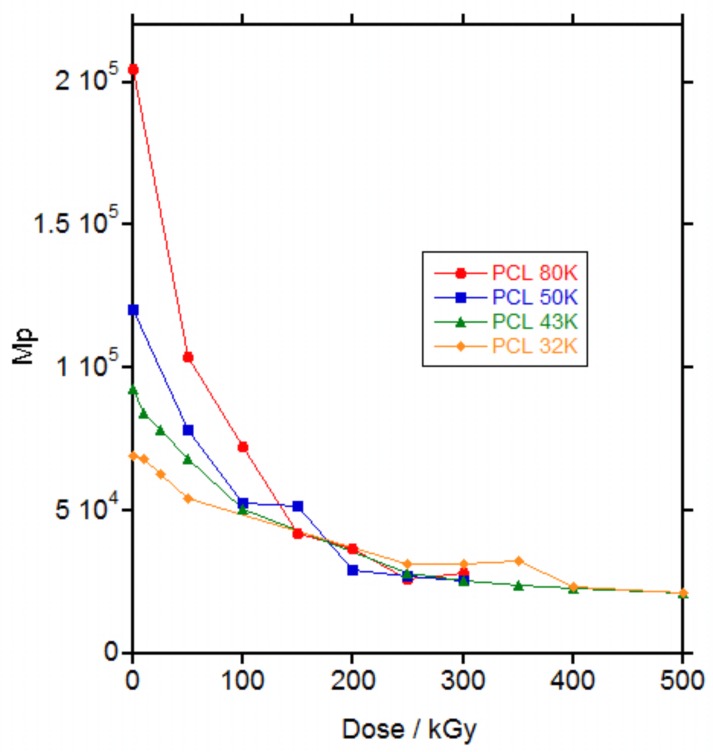
Molecular weight on the maximum of the size exclusion chromatography (SEC) curve (*M*_p_) vs. dose for the irradiated PCLs. The molecular weight corresponds to the whole sample at doses below gelation (below 100 kGy for PCL 80 K and below 150 kGy for PCL 50 K, PCL 43 K and PCL 32 K) and to the soluble part of the sample above gelation.

For all the PCLs, irradiation produced a decrease in the molecular weight on the maximum (*M*_p_) of the SEC curve (Figure 3) and an increase in the polydispersity of the polymer reaching the total exclusion limit of the columns (Figures S7–S10 in the Supporting Information file). The increase in the polydispersity reached a maximum at a dose of 50, 100, 100 and 250 kGy for PCL 80, 50, 43 and 32 K, respectively, which is coincident with the maximum dose for gelation (58, 89, 127 and 204 kGy respectively) and then decreased for higher doses when gel was formed.

The increase in polydispersity with the increase in dose until gelation was reached has been already described in literature for PCLs irradiated with gamma rays [6,9]. Generally, an increase in molecular weight was described until gelation [8,9,13] followed by a decrease in molecular weight for the soluble part of the material after gelation [12] and only in one work a decrease in molecular weight was found until gel [11] as seen in Figure 3. This apparent contradiction with the results for the PCLs in this study was solved when the weight average molecular weight (*M*_w_) versus dose was plotted, as in Figure S11 in the Supporting Information file. Whereas *M*_p_ decreased continuously with the increase in dose, Figure 3, *M*_w_ increased with the increase in dose until gelation and then decreased in the soluble part of the material, Figure S11 in the Supporting Information file. Thus, until gelation, branching produced by irradiation leading to an increase in *M*_w_ and polydispersity, dominated over the decrease in molecular weight produced by scission. After gelation, only the fragments produced by scission are soluble and could be measured by SEC and a decrease in molecular weight was found as a consequence of the increase in the scission.

### 3.4. Proton NMR Spectra of Irradiated PCL

Proton NMR was used to determine the chemical species produced by irradiation. In Figure S12 in the Supporting Information file the spectra for PCL 50 K non-irradiated and for the soluble part of the PCL 50 K irradiated at 500 and 1000 kGy are presented. Besides the main peaks at 4.05, 2.30, 1.64 and 1.37 ppm for high molecular weight PCL, some new small peaks appeared at 3.64, 2.37 and 0.89 ppm that increased their intensity with the increase in dose, as seen in Figure S13 in the Supporting Information file. Triplets at 3.64 and 2.37 ppm were assigned to methylenes next to hydroxyl (–CH_2_–OH) and carboxylic groups (–CH_2_–COOH), respectively, in agreement with the triplets found for α-hydroxyl-*ω*-(carboxylic acid) polycaprolactone [26]. Multiplet at 0.89 ppm was assigned to protons in a saturated hydrocarbon chain. The assignations were confirmed by derivatization of the samples with trifluoroacetic anhydride. Upon derivatization, triplets at 3.64 and 2.37 ppm shifted to 4.34 and 2.63 ppm respectively, as already seen for α-hydroxyl-*ω*-(carboxylic acid) polycaprolactone [26,27], whereas the multiplet at 0.89 ppm remained unchanged (see Figure S14 in the Supporting Information file.

**Figure 4:**
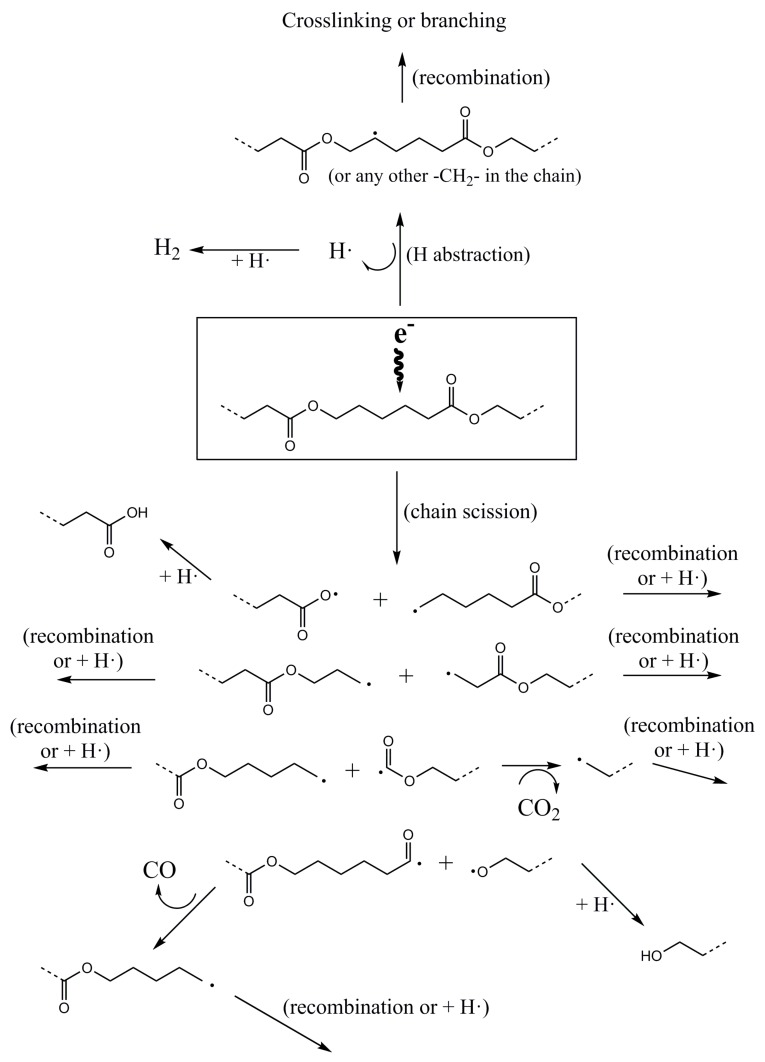
Proposed mechanism of degradation of PCL by electron-beam irradiation.

Studies on the effect of electron-beam on PCL found in literature demonstrated, by electron paramagnetic resonance (EPR), the existence of radical species of the type –CH_2_–CH_2_–ĊH–COO– and/or –COO–ĊH–CH_2_–CH_2_– [13]. In addition, the analysis of the gases released when PCL was irradiated with gamma rays showed the formation of H_2_, CO and CO_2_, with a molar ratio of 2.1, 1.0, 1.1 respectively [7]. From these data and the formed species identified by NMR, a reaction mechanism of PCL with electron beam is proposed as seen in Figure 4.

Electron beam irradiation of PCL can produce radicals in the polymer chain by hydrogen abstraction and chain scission at any bond within the polymer chain, as seen in Figure 4. When hydrogen abstraction takes place, the generated carbon radical can recombine with another radical from another chain leading to a branching point that increases the molecular weight and ultimately to a network. The H_2_ released when PCL was irradiated with gamma rays, coming from the recombination of two hydrogen radicals [7], proved that hydrogen abstraction is produced in PCL when irradiated with high energy radiation.

Chain scission can take place at any bond in the PCL chain. When the –COO–CH_2_– bond is broken, the oxygen radical can recombine with a hydrogen radical to produce a carboxylic group, as detected by proton NMR, whereas the carbon radical can recombine with another carbon radical to produce branching or with a hydrogen radical to produce a saturated chain end. When the –CH_2_–CH_2_– bond is broken, the carbon radicals can recombine with another carbon radical or a hydrogen radical. When the –CH_2_–COO– bond is broken, the carbon radical can recombine with another carbon radical or a hydrogen radical and the carboxylic radical can decompose to a carbon radical (that can recombine with another carbon radical or a hydrogen radical) with the release of CO_2_, as detected for PCL irradiated with gamma rays [7]. And, when the –CO–O– bond is broken, the carbonylic radical can decompose to a carbon radical (that can recombine with another carbon radical or a hydrogen radical) with the release of CO, as detected for PCL irradiated with gamma rays [7] and the oxygen radical can recombine with a hydrogen radical to produce a hydroxyl group, as detected by proton NMR.

The molar ratio for CO and CO_2_, of 1.0 and 1.1 respectively, found for PCL irradiated with gamma rays [7], would be interpreted following Figure 4 as a similar probability of scission for the –CH_2_–COO– bond (release of CO_2_) and the –CO–O– bond (release of CO).

Proton NMR signals from Figure S14 were integrated to evaluate the relative abundance of the produced species. Signal at 4.05 ppm from the methylenes next to the oxygen of the PCL ester group (–CH_2_–O–CO–) was taken as the internal reference. Signals at 4.34, 2.63 and 0.89 ppm for methylenes next to hydroxyl (–CH_2_–OH), methylenes next to carboxylic groups (–CH_2_–COOH) and protons in a saturated hydrocarbon chain respectively, were integrated and ratioed to the internal reference. Results are listed in Table 1.

The most abundant species generated by electron beam irradiation were carboxylic groups whereas the hydroxyl groups and the saturated hydrocarbon bonds had similar presence. From these results, it could be deduced that irradiation breaks preferentially the –COO–CH_2_– bond as shown in Figure 4.

The ratio of the groups does not vary significantly with the increase in dose thus it seems that the mechanism of degradation is independent of the dose.

From the integrals of the signals and by considering that carboxylic and hydroxyls groups are the only terminal groups in the chains and that the chains are linear (which is not true for the irradiated materials because branching was produced by irradiation), the molecular weight of the chains can be calculated. In Table 1, *M*_n_ calculated by NMR and the changes in *M*_n_ with irradiation as measured by NMR and SEC, are presented. As it can be seen, *M*_n_ for the non-irradiated PCL 50 K was close to 50,000 g∙mol^−1^. After irradiation, *M*_n_ of the soluble part of the polymer decreased strongly with calculated values quite similar to *M*_n_ as calculated by SEC despite the facts that branching was not taken into account and the differences in the experimental techniques.

### 3.5. Thermal Properties

Changes in thermal properties with irradiation doses up to 200 kGy for PCL 50 K and up to 300 kGy for PCL 80 K were measured by DSC. The upper limit for the irradiation dose was chosen after the catastrophic decay in mechanical properties was reached, as it will be shown later. Samples were measured by duplicate to estimate the dispersion of the results.

The general shape of the DSC traces was the same for all the samples. In Figure S15 in the Supporting Information file, the DSC traces for PCL 80 K irradiated at 200 kGy are shown as an example. In the first heating, a broad endotherm due to the melting of the PCL crystals was observed. On cooling and exotherm demonstrated partial PCL crystallization and in the second heating, a *T*_g_ due to the amorphous part of the PCL was registered at low temperatures followed by a melting endotherm of the PCL crystals at higher temperatures.

In Figures S16–S19 in the Supporting Information file the changes in melting point (*M*_p_), melting enthalpy, glass transition middle point (*T*_g_) and crystallization enthalpy with dose can be seen and in Figure 6 the changes in crystallization temperature (*T*_c_) with dose are represented.

No significant changes were observed for the melting point (Figure S16) in the first heating. In the second heating cycle a slight trend to decrease was observed although the dispersion of the data is high (Figure S16). For the melting enthalpy (Figure S17), related to percentage of crystallinity, a slight trend to increase with the increase in dose could be guessed in both heating cycles but the dispersion of the data was too high to consider the trend as significant.

For the glass transition temperature (Figure S18) and the crystallization enthalpy (Figure S19), a slight trend to increase their values with the increase in dose can be observed although the big dispersion of the data again masks the trend and makes it statistically not significant. For the glass transition temperature, the change was very small, within a range of 3 °C and although a trend to increase with the increase in dose could be appreciated, the dispersion of the data makes the trend statistically not significant.

**Figure 5:**
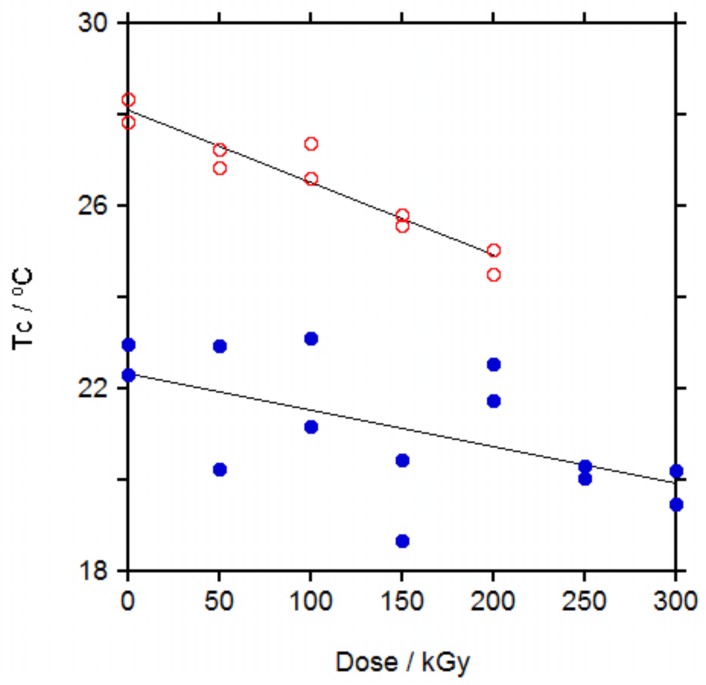
Crystallization temperature (*T*_c_) for PCL 50 K (open symbols) and for PCL 80 K (filled symbols).

Crystallization temperature (*T*_c_) showed the clearest trend, Figure 5, with a significant decrease in *T*_c_ with the increase in dose for PCL 50 K. The same trend was found for PCL 80 K but with a higher dispersion in the data.

These trends for PCL are similar to those found for other semicrystalline polymers. For PE irradiated up to 233 kGy, *M*_p_ and crystallinity remained almost unchanged and *T*_c_ decreased slightly with the increase in dose [28,29], similar to PCLs in this study. These results were explained by the formation of crosslinking and branching taking place mainly on the amorphous regions and on the boundaries of crystallites, that affects the melting point but more strongly the crystallization temperature as the crosslinks disturbs the formation of crystals on cooling, leading to a delay on the crystallization. For PA-6, when irradiated up to 400 kGy, *M*_p_ and *T*_c_ decreased slightly, more *T*_c_ than *M*_p_ [30], thus it seems that irradiation affects to the boundaries of crystallites of PA-6 more than to PE and PCL. Changes in *T*_g_ for PA-6 were rather small, as for PCLs, due to the similar effect of the crosslinking leading to an increase in *T*_g_ and the chain scission leading to a decrease in *T*_g_. The same was found for the crystallinity in PA-6, were the decrease in crystallinity due to crosslinking was compensated by the increase in crystallinity due to the shorter chains produced by chain scission and overall crystallinity did not change significantly with the increase in dose.

When the effect of irradiation with ionizing radiation on the thermal properties of PCL was revised, it was found that the melting point decreased with the increase in dose [6,8,10], in agreement with our results for the melting point in the second heating cycle. For the crystallinity, when PCL was irradiated with gamma rays, it was found that it increased with the increase in dose up to a dose of 500 kGy and then decreased at higher doses [6], it slightly increased after irradiation at 35 and then slightly decreased after irradiation at 65 kGy and it increased continuously up to a dose of 300 kGy [4]. When PCL was irradiated with electron beam up to 200 kGy, crystallinity increased continuously with the increase in dose [16]. This general trend to increase crystallinity with the increase in dose is in agreement with our results (Figure S17) and the high dispersion of the data observed could account for the apparent initial increase and posterior decrease described in reference 6. Only in one work it was described that crystallinity decreased with the increase in dose when PCL was irradiated with electron beam up to 1000 kGy but in this work crystallinity was evaluated from WAXD spectra [18]. DSC measurements are more sensitive than WAXD spectra for the determination of the crystallinity of a material and quantification of crystallinity from DSC curves is more precise than quantification from WAXD spectra thus we consider that the decreasing trend in this work is due to the limitation of the technique. The change in crystallization temperature, *T*_c_, was measured only in two works. In one of them, when PCL was irradiated with gamma rays up to 500 kGy, *T*_c_ decreased with the increase in dose [8], in agreement with our results, whereas in the other work, when PCL was irradiated with electron beam up to 250 kGy, *T*_c_ increased with the increase in dose, in disagreement with our results [16].

Similar to PE and PA-6, thermal properties changes in PCL irradiated with gamma rays are explained by crosslinking reactions occurring between chains in their relaxed state, that is, in the amorphous phase, with only little effect on the solid crystallites. Thus, *M*_p_ in the first heating cycle would not be affected but after melting, crosslinks would hinder crystal growth, decreasing *T*_c_ (slower crystallization) and decreasing *M*_p_ in the second heating run (smaller size of the crystals). At the same time, chain scission produces shorter chains that can crystallize better than longer chains and would account for the slight increase in the crystallinity with the increase in dose.

### 3.6. Mechanical Properties

Mechanical properties were measured for PCL 32, 43, 50 and 80 K after irradiation by electron beam up to 250 kGy and by gamma rays up to 200 kGy.

The shape of the stress-strain curve remained the same irrespective of the irradiation dose. The effect of increasing the dose was the decrease of the strain that led to a decrease in the tensile strength.

**Figure 6:**
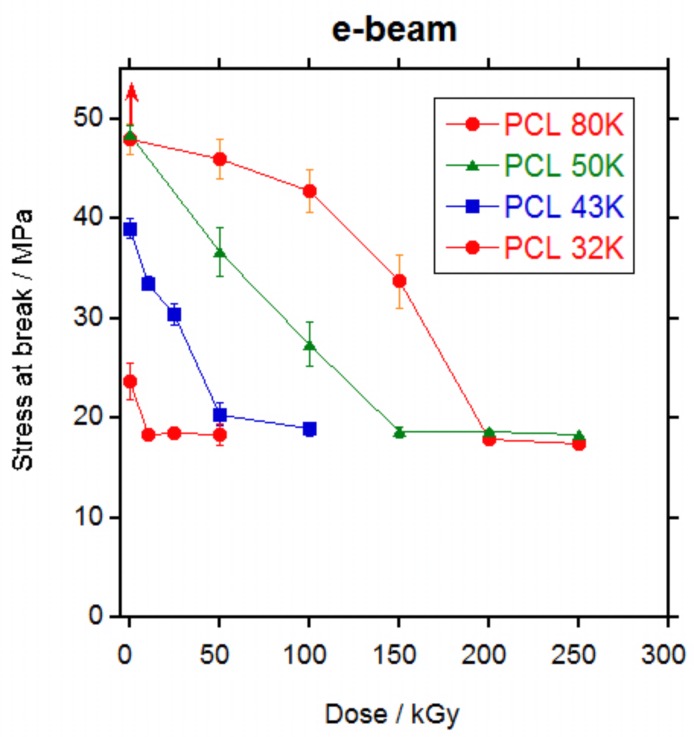

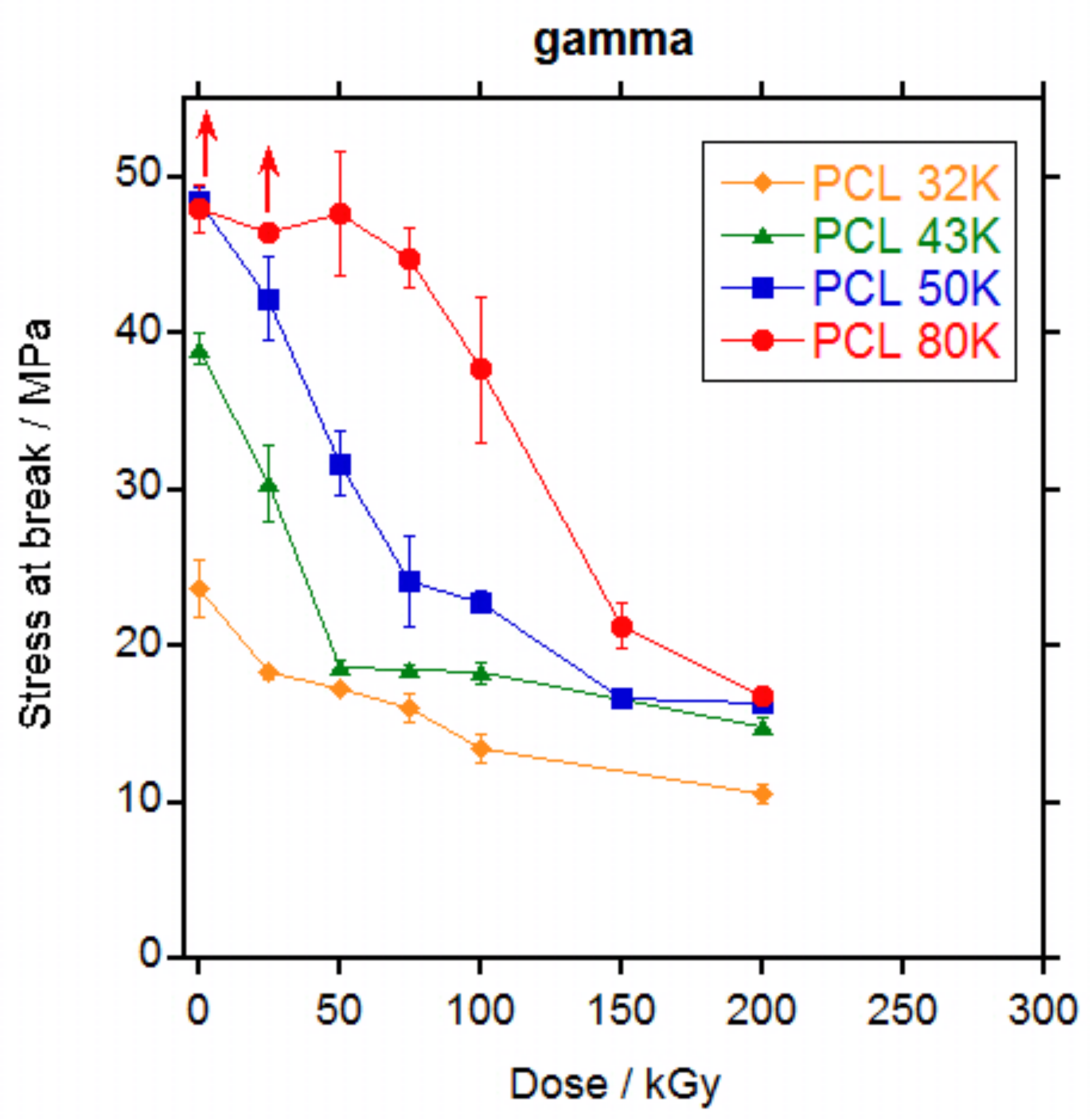
Stress at break values for PCL 32, 43, 50 and 80 K irradiated at different doses by electron beam (top) and by gamma rays (bottom).

**Figure 7:**
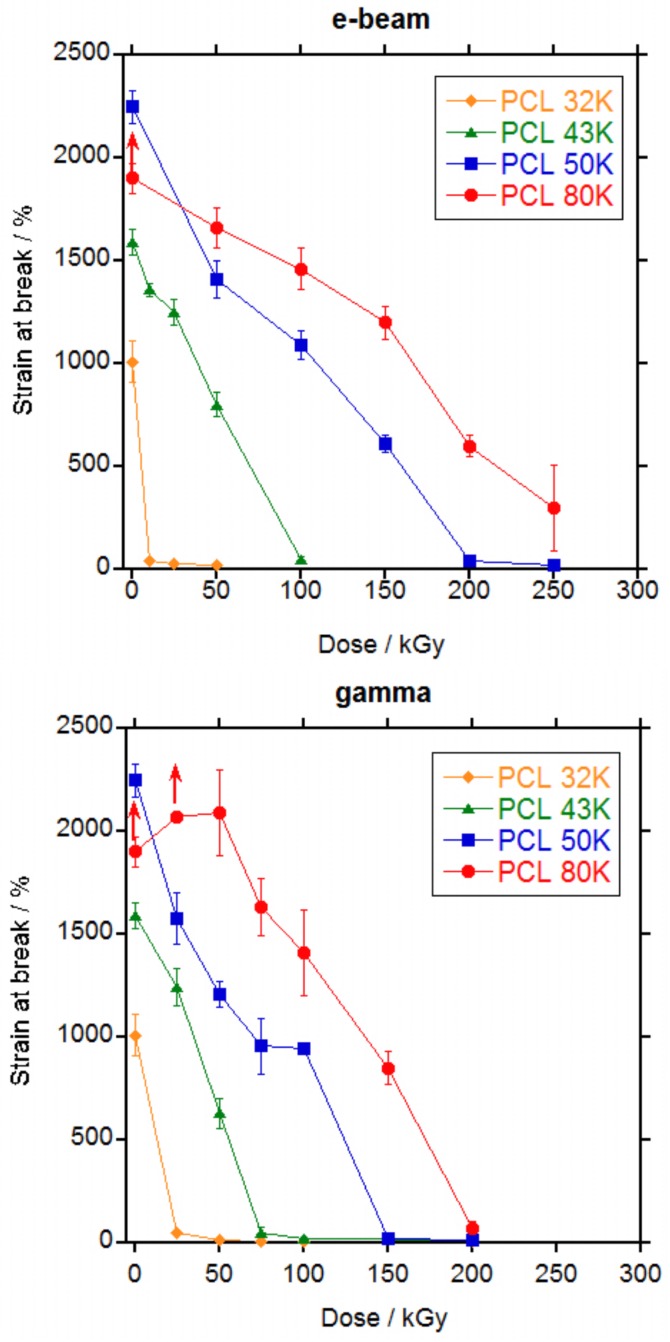
Strain at break values for PCL 32, 43, 50 and 80 K irradiated at different doses by electron beam (top) and by gamma rays (bottom).

In Figure 6 and Figure 7, the values for tensile stress at break and tensile strain at break versus dose are represented, respectively and in Tables S1 and S2 in the Supporting Information file are listed. For non-irradiated PCL 80 K, as mentioned previously, specimens escaped from the grip before rupture. The same happened to PCL 80 K irradiated 25 kGy by gamma rays. For these samples, the values represented in Figure 6 and Figure 7 are the mean values recorded until the specimens escaped and the real value for tensile stress and strain will be higher, as indicated by the arrows in the Figures.

A continuous decrease in the stress at break (Figure 6) and in the strain at break (Figure 7) was observed with the increase in dose for all PCLs. For PCL 32 K, strain dropped sharply to below 50% at the lowest dose (10 kGy for electron beam and 25 kGy for gamma rays); for PCL 43 K the strain dropped to less than 50% at a dose of 100 kGy for electron beam and 75 kGy for gamma rays; for PCL 50 K the strain dropped to less than 50% at a dose of 200 kGy for electron beam and 150 kGy for gamma rays; and for PCL 80 K the strain still did not drop to less than 50% at a dose of 250 kGy for electron beam and dropped to slightly above 50% at a dose of 200 kGy for gamma rays. From the data, it is clear that mechanical properties are adversely affected by ionizing radiation and that the retention of the properties is better when the molecular weight of the PCL is higher. When electron beam and gamma rays are compared, it can be seen that gamma rays produces a slightly higher damage. The slight difference in the results for electron beam and gamma rays is probably due to the differences in dose rate. At high dose rates such as for electron beam, the number of radicals formed by unit time is high and if they do not have enough time to diffuse they would recombine without producing any effect. At low dose rates such as gamma rays, the number of radicals is low and it is more difficult for them to recombine thus they will diffuse and produce crosslinking or scission [31].

It is well known that crystallinity is one of the primary factors that influence the mechanical properties of polymers [32]. However, as seen on Figure S17 in the Supplementary information file, crystallinity does not change significantly with irradiation dose thus changes in the mechanical properties are not due to changes in crystallinity and it is entirely due to the effect of the crosslinking in the amorphous part of the polymer.

When literature data were reviewed, different behaviours were found. In all cases, strain at break decreased with the increase in dose [6,7,15,17] as found in this work except for one case where a slight increase in strain at break is found for a PCL of unknown molecular weight after irradiation with gamma rays at a 30.8 kGy dose [3]. For the stress at break, however, an increase in tensile stress was found when PCL was irradiated with electron beam [15,17] or gamma rays [3,7] up to 160–200 kGy followed by a decrease at higher doses. This is not the case for the results in this work, where a continuous decrease in stress at break was found with the increase in dose either with electron beam or gamma rays, without significant differences in between both ionizing radiations.

The decrease in mechanical properties is explained by the scission of the chains in the amorphous phase that dominates over the crosslinking. Scission of the chains connecting the crystalline domains, that are the load bearing elements in the material, leaves the crystalline domains untied, leading to the decrease in the elongation and the tensile strength, as already stated by other authors [6].

In Figures S20 and S21 in the Supplementary information file, the retention of the mechanical properties for PCL 50 K and PCL 80 K are compared with that for other semicrystalline polymers, PA-6 [30] and PET [33] and for an almost completely amorphous aliphatic polyurethane [34]. As it can be seen, irradiation affects much more to PCL than to the other materials and therefore is the material with the lowest resistance to ionizing radiation.

At sterilization doses, PCL 32 K would become very fragile and could not be used in a medical implant if a minimum level of mechanical properties should be required. PCL 43K would become fragile if sterilized at values above 50 kGy, whereas for values below 50 kGy it would retain part of its mechanical properties and depending on the application, PCL 43 K could still be useful after sterilization. For PCL 50 K sterilization doses produce a decay in properties but the level of retention of the mechanical properties is still fair, better than PCL 43 K. PCL 80 K showed by far the best retention of mechanical properties after irradiation at sterilization doses, with very high values of stress and strain even after irradiation at 100 kGy. From these results, it is clear that the higher the molecular weight of the PCL the better the retention of the mechanical properties after irradiation. And that for a medical implant with a minimum requirement of mechanical properties to be sterilized with ionizing radiation, it would be necessary to use a PCL of at least 50,000 g·mol^−1^ molecular weight.

## 4. Conclusions

Irradiation of PCLs of different molecular weights with ionizing radiation produced a crosslinked material with a gel content that increased with the increase in dose and with the increase in the initial molecular weight of the PCL.

Charlesby-Pinner analysis demonstrated that both crosslinking and chain scission took place, without a significant predominance of one mechanism over the other. Minimum dose for gelation decreased with the increase in the molecular weight of the PCL from 255 to 58 kGy when PCL molecular weight increased from 25,000 to 80,000 g·mol^−1^. Crosslinking efficiency slightly decreased from 1.22 to 0.97 when PCL molecular weight increased, probably due to the fitting of the data to a straight line instead of a curve.

Before gelation, irradiation generated branching that increased the molecular weight as demonstrated by SEC. Analysis by proton NMR of the soluble part of the PCLs demonstrated the formation of hydroxyl, carboxylic and saturated aliphatic species, consistent with the rupture of any bond in the PCL chain with preference for the scission of the –COO–CH_2_– bond.

Irradiation produced a significant decrease in crystallization temperature. For the rest of the thermal properties, the changes (melting point decrease; crystallinity and *T*_g_ increase) were of low significance due to the high dispersion of the data. Changes in melting point, *T*_g_ and crystallization temperature were explained by the difficulty for the rearrangement of the chains produced by the crosslinks and the increase in crystallinity by the presence of short chains due to scission.

Mechanical properties were strongly affected by irradiation. As a consequence of the chain scission in the amorphous phase, stress at break and strain at break were continuously reduced with the increase in dose until a fragile material was obtained, irrespective of the initial molecular weight of the PCL. When the molecular weight of the PCL increased, the dose needed to obtain a fragile material increased. For PCL, molecular weight below 50,000 g·mol^−1^, PCL becomes fragile already at sterilization doses. A slightly more deleterious effect of gamma rays was found when compared to electron beam. PCL demonstrated to be less resistant to ionizing radiation than other polymers semicrystalline polymers such as PA-6 or PET.

## Figures and Tables

**Table 1:** Calculations from proton nuclear magnetic resonance (NMR) spectra for PCL 50 K non-irradiated and irradiated at 500 and 1000 kGy.

Dose/kGy	0	500	1000
CH_2_OH/Ref	0.00427	0.0124	0.0245
CH_2_COOH/Ref	0	0.0308	0.0581
0.89 ppm/Ref	0	0.0137	0.0249
*r* CH_2_COOH/CH_2_OH	-	2.47	2.37
*r* 0.89 ppm/CH_2_OH	-	1.10	1.02
*M*_n_ (NMR)	53,500	5530	2960
%*M*_n_ (NMR)	100	10	6
%*M*_n_ (SEC)	100	17	8

## Supplementary Materials

The following are available online at http://www.mdpi.com/2073-4360/10/4/397/s1, Figure S1: Molecular weight in the peak maximum (*M*_p_) of the SEC curve versus molecular weight as reported by the supplier (*M*_n_) of polycaprolactones, Figure S2: Tensile stress-strain curves for the polycaprolactones, Figure S3: Tensile stress and strain results for the polycaprolactones, Figure S4: Crystallinity percentage for the polycaprolactones in the pellets and after recrystallization from the melt for 30 min and 10 days, Figure S5: Calculated crosslink efficiency (*q*_0_/*p*_0_) versus PCL molecular weight, Figure S6: Calculated minimum dose for gelation (*d*_0_) versus PCL molecular weight, Figure S7: SEC curves for PCL 80 K, Figure S8: SEC curves for PCL 50 K, Figure S9: SEC curves for PCL 43 K, Figure S10: SEC curves for PCL 32 K, Figure S11: Weight average molecular weight (*M*_w_) versus dose for the irradiated PCLs, Figure S12: Proton NMR spectra for PCL 50 K non irradiated (black line) and for the soluble part of the PCL 50 K irradiated at 500 (red line) and 1000 kGy (blue line), Figure S13: Expanded view of the spectra in Figure S12, Figure S14: Proton NMR spectra for PCL 50 K non irradiated (black line) and for the soluble part of the PCL 50 K irradiated at 500 (red line) and 1000 kGy (blue line) derivatized with trifluoroacetic anhydride, Figure S15: DSC traces for PCL 80 K irradiated with electron beam at a dose of 200 kGy, Figure S16: Melting point (*M*_p_) for PCL 50 K (in red) and for PCL 80 K (in blue) in the first heating run (open symbols) and in the second heating run (filled symbols), Figure S17: Melting enthalpy for PCL 50 K (in red) and for PCL 80 K (in blue) in the first heating run (open symbols) and in the second heating run (filled symbols), Figure S18: Glass transition temperature (*T*_g_) for PCL 50 K (in red) and for PCL 80 K (in blue), Figure S19: Crystallization enthalpy for PCL 50 K (in red) and for PCL 80 K (in blue), Figure S20: Retention of the stress at break versus dose for PCL 50 K (red circles), PCL 80 K (blue circles), PA-6 (green squares), PET (black triangles) and for an aliphatic polyurethane (magenta diamonds), Figure S21: Retention of the strain at break versus dose for PCL 50 K (red circles), PCL 80 K (blue circles), PA-6 (green squares), PET (black triangles) and for an aliphatic polyurethane (magenta diamonds), Table S1: Values for tensile stress at break (*T*_s_) and strain at break (ε_b_) of PCLs when irradiated with electron beam at several doses, Table S2: Values for tensile stress at break (*T*_s_) and strain at break (ε_b_) of PCLs when irradiated with gamma rays at several doses.
