# Supplementary material for: Effect of Ionizing Radiation on the Chemical Structure and the Physical Properties of Polycaprolactones of Different Molecular Weight
Source: Polymers (Basel). 2018 Apr 3;10(4):397. doi: 10.3390/polym10040397 (PMC6415462; doi:10.3390/polym10040397)
Supplement: Supplementary file 1 [file polymers-10-00397-s001.pdf]

polymers

Effect of Ionizing Radiation on the Chemical Structure and the Physical Properties of Polycaprolactones of Different Molecular Weight

Rodrigo Navarro [1], Guillermina Burillo [2], Esbaide Adem [3] and Angel Marcos-Fernández [1,*]

[1] Instituto de Ciencia y Tecnología de Polímeros (CSIC), Juan de la Cierva 3, 28006 Madrid, Spain; rnavarro@ictp.csic.es
[2] Instituto de Ciencias Nucleares, Universidad Nacional Autónoma de México, Circuito Exterior, Ciudad Universitaria, 04510 Ciudad de México, México; burillo@nucleares.unam.mx
[3] Instituto de Física, Universidad Nacional Autónoma de México, Circuito Exterior, Ciudad Universitaria, 04510 Ciudad de México, México; esbaide@fisica.unam.mx
* Correspondence: amarcos@ictp.csic.es; Tel.: +34-912-587-555

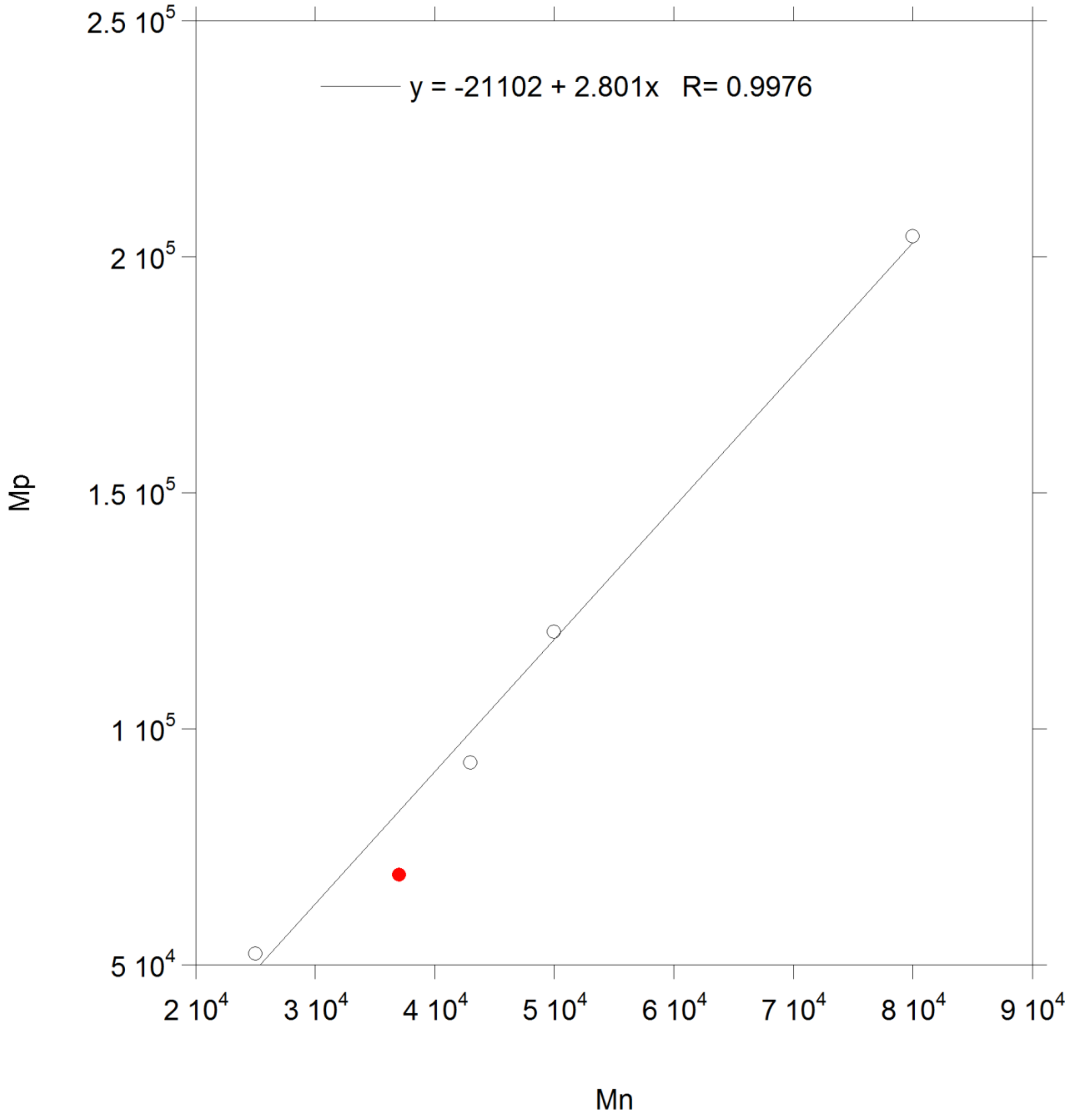

Figure S1. Molecular weight in the peak maximum (M_P) of the size exclusion chromatography (SEC) curve vs molecular weight as reported by the supplier (M_n) of polycaprolactones.

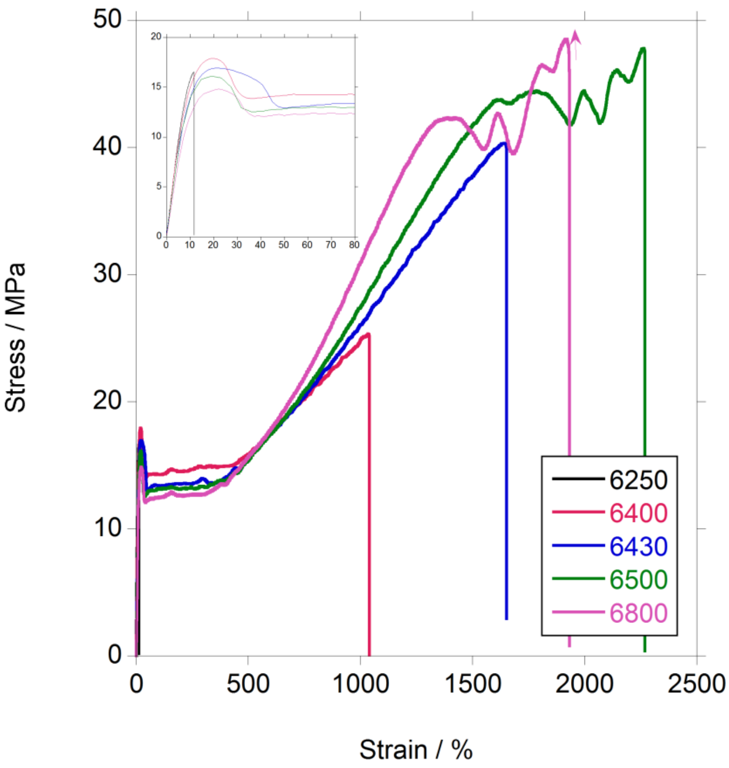

Figure S2. Tensile stress-strain curves for the polycaprolactones.

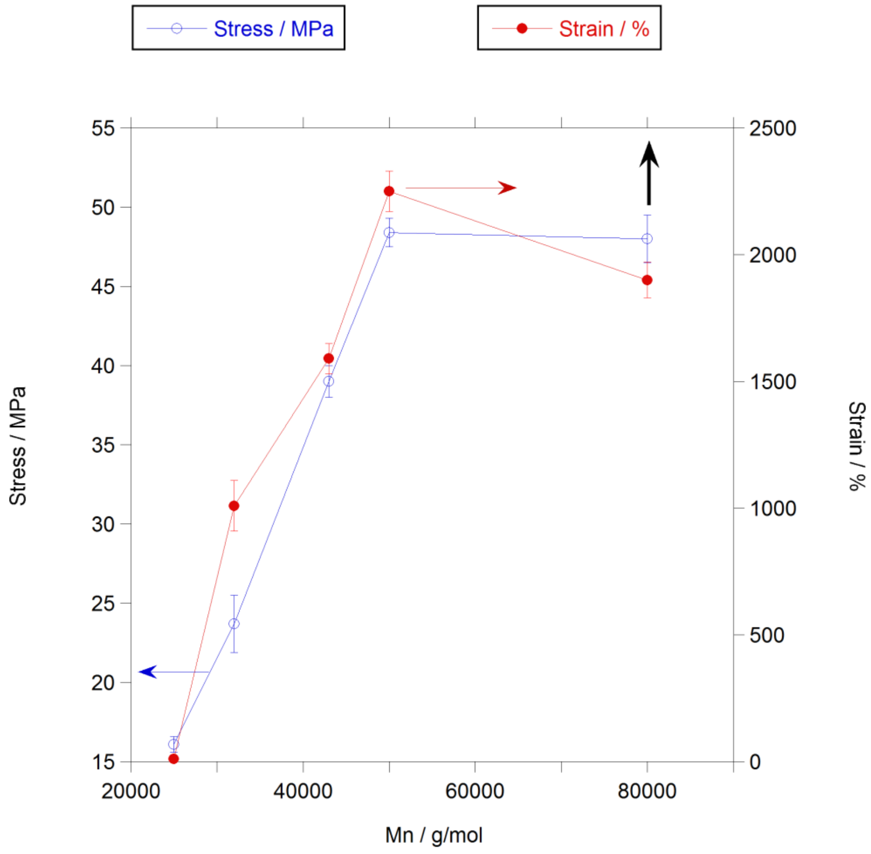

Figure S3. Tensile stress and strain results for the polycaprolactones.

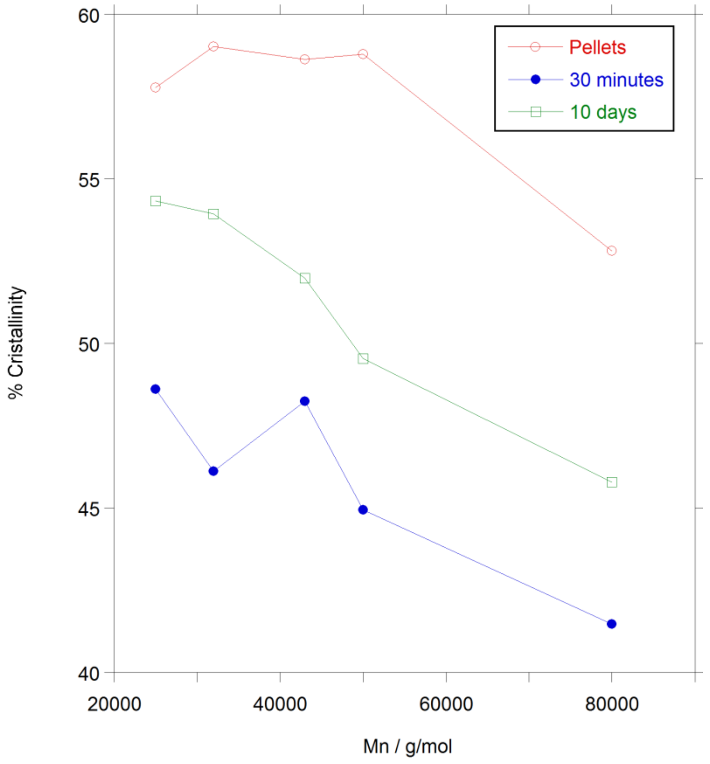

Figure S4. Crystallinity percentage for the polycaprolactones in the pellets and after recrystallization from the melt for 30 min and 10 days.

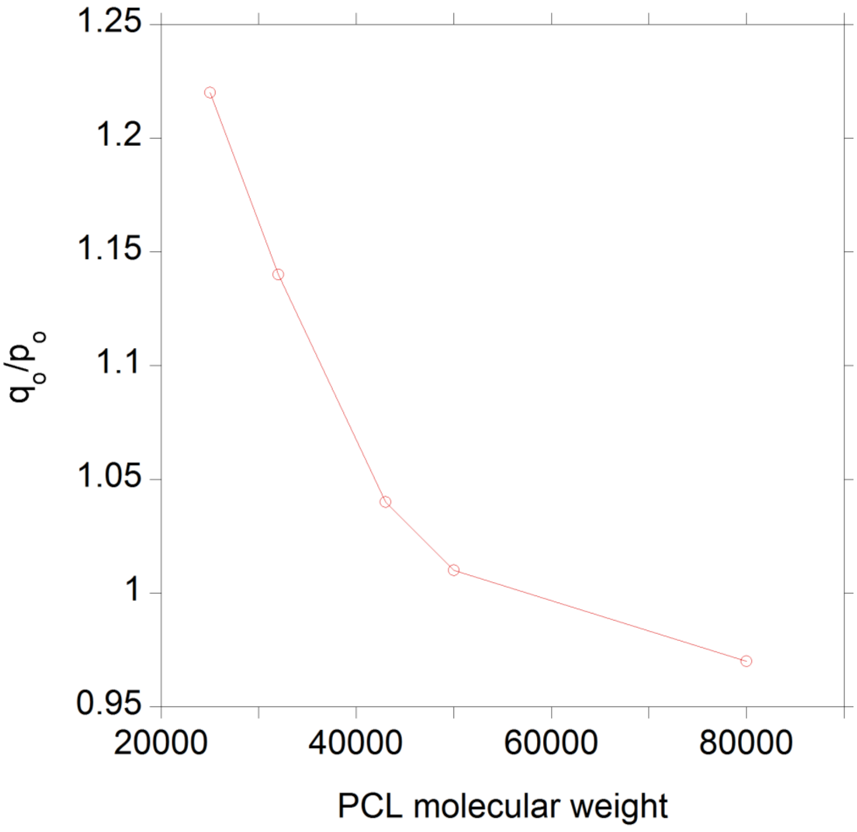

Figure S5. Calculated crosslink efficiency (q_0/p_0) vs PCL molecular weight.

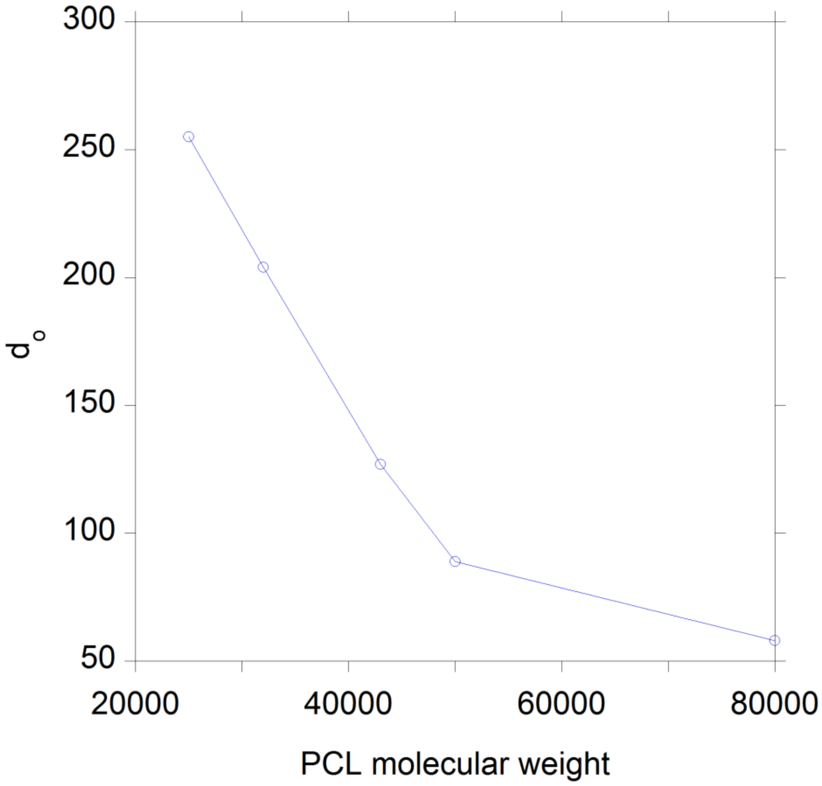

Figure S6. Calculated minimum dose for gelation (d_0) vs PCL molecular weight.

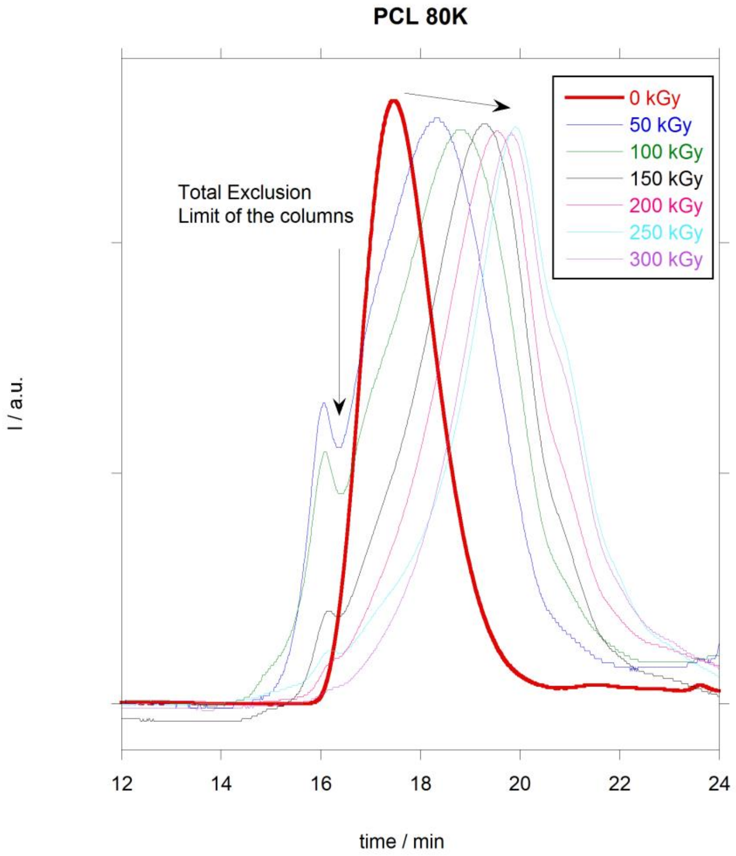

Figure S7. SEC curves for PCL 80 K.

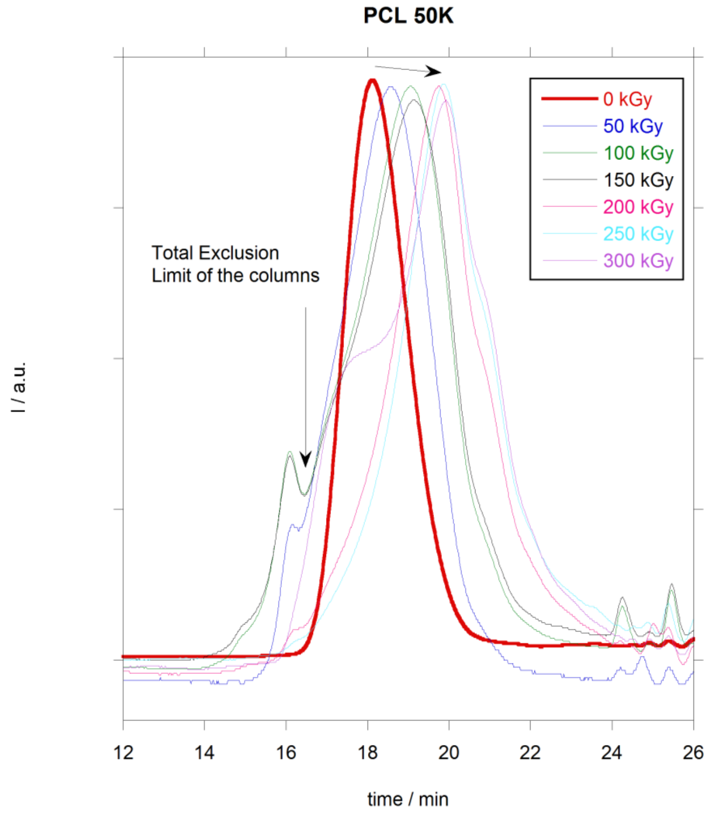

Figure S8. SEC curves for PCL 50 K.

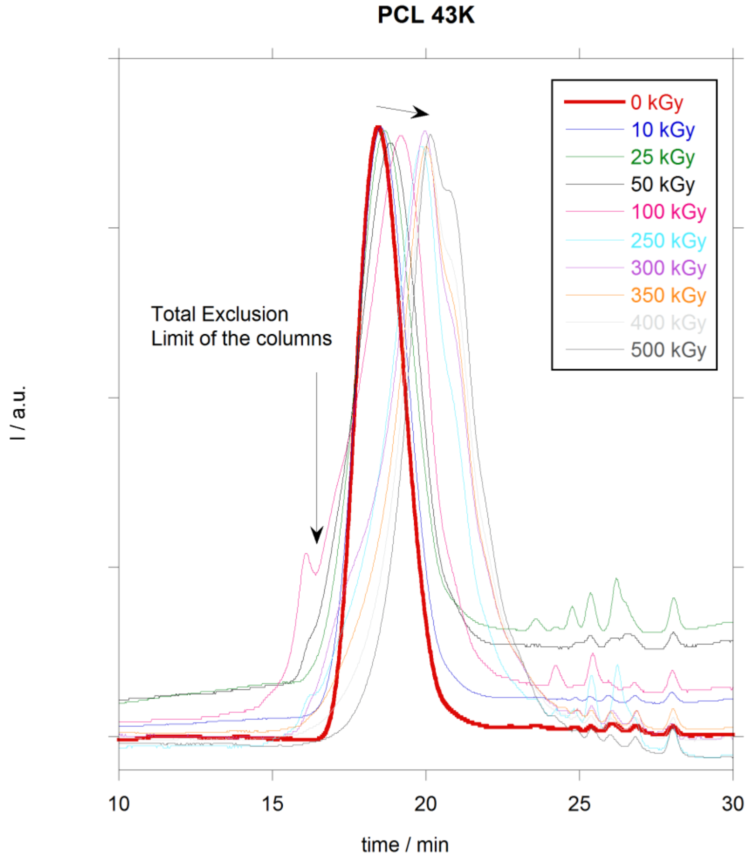

Figure S9. SEC curves for PCL 43 K.

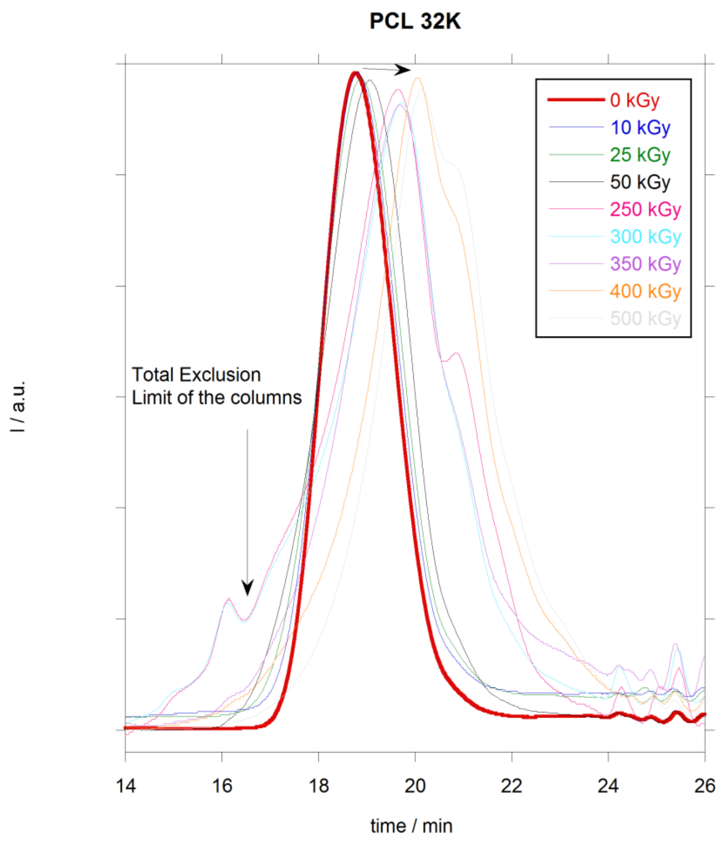

Figure S10. SEC curves for PCL 32 K.

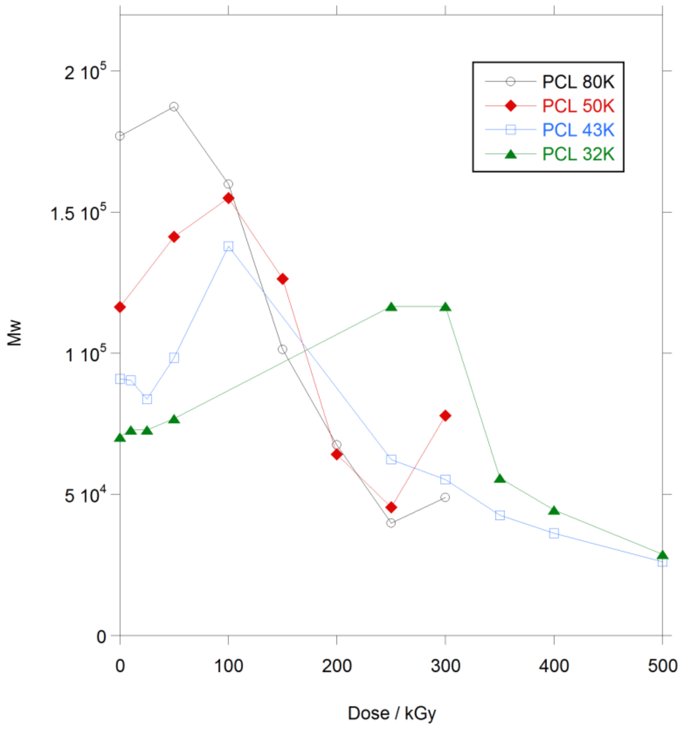

Figure S11. Weight average molecular weight (M_w) vs dose for the irradiated PCLs.

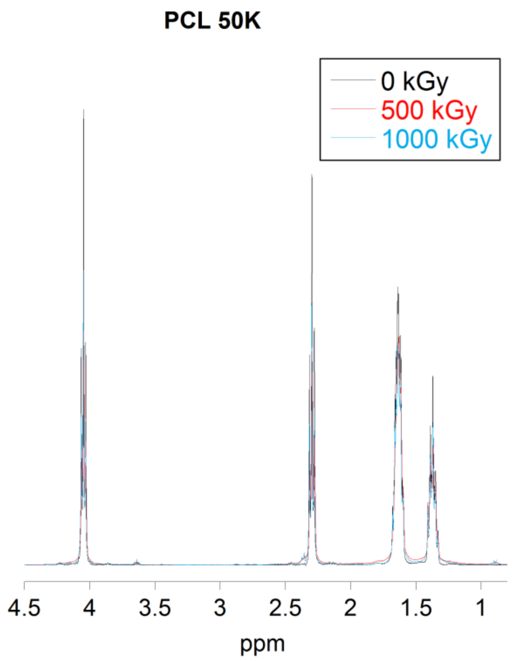

Figure S12. Proton nuclear magnetic resonance (NMR) spectra for PCL 50 K non irradiated (black line) and for the soluble part of the PCL 50 K irradiated at 500 (red line) and 1000 kGy (blue line).

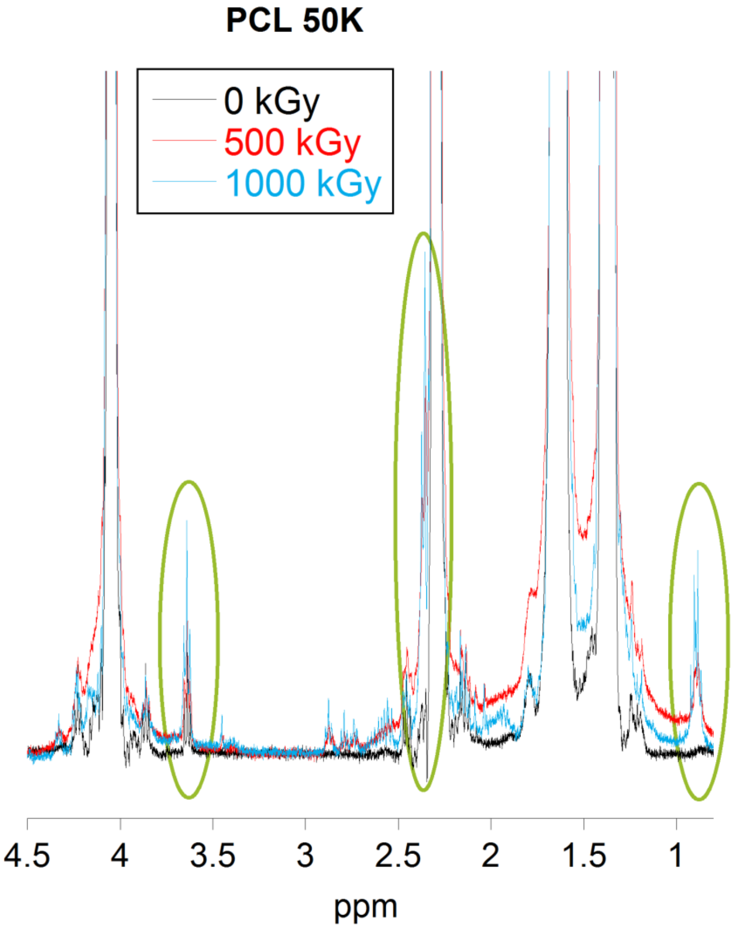

Figure S13. Expanded view of the spectra in Figure S12.

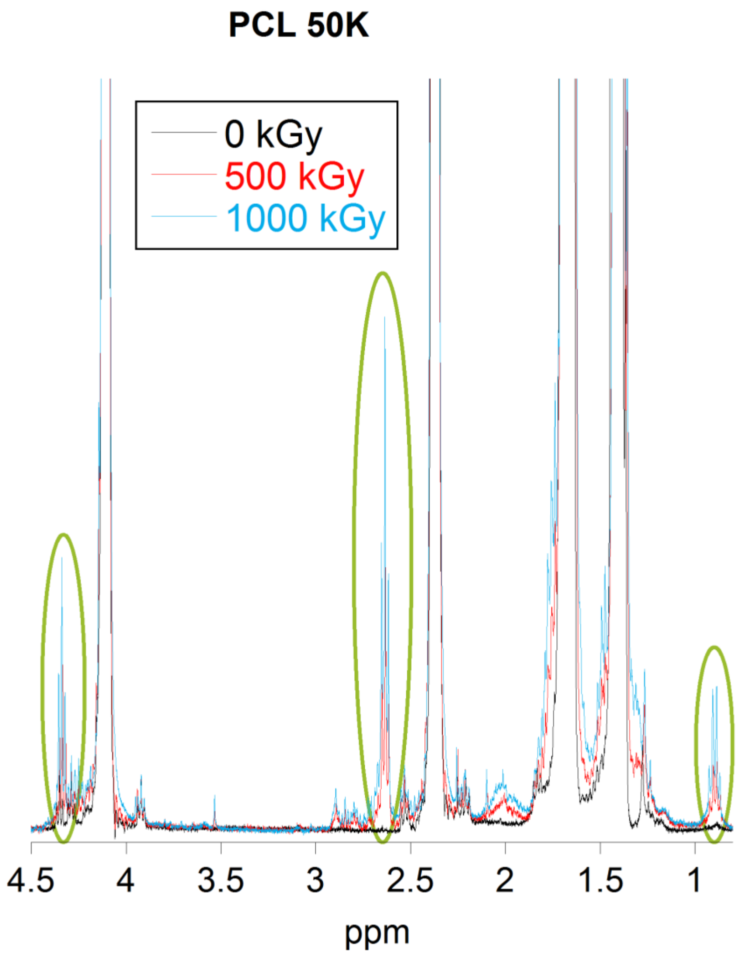

Figure S14. Proton NMR spectra for PCL 50 K non irradiated (black line) and for the soluble part of the PCL 50 K irradiated at 500 (red line) and 1000 kGy (blue line) derivatized with trifluoroacetic anhydride.

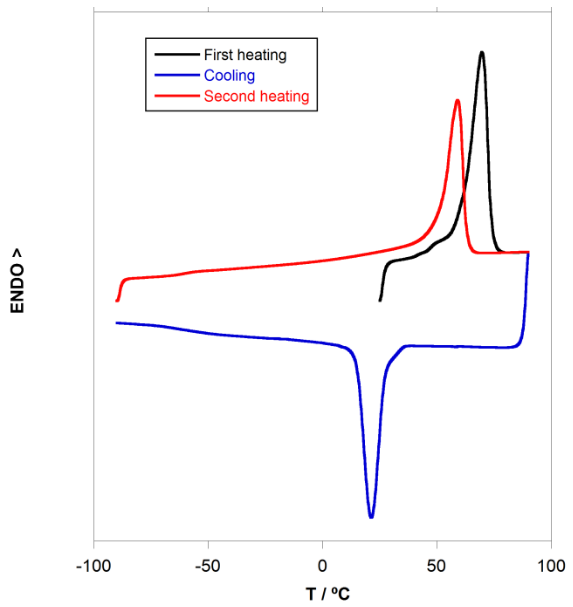

Figure S15. Differential Scanning Calorimetry (DSC) traces for PCL 80 K irradiated with electron beam at a dose of 200 kGy.

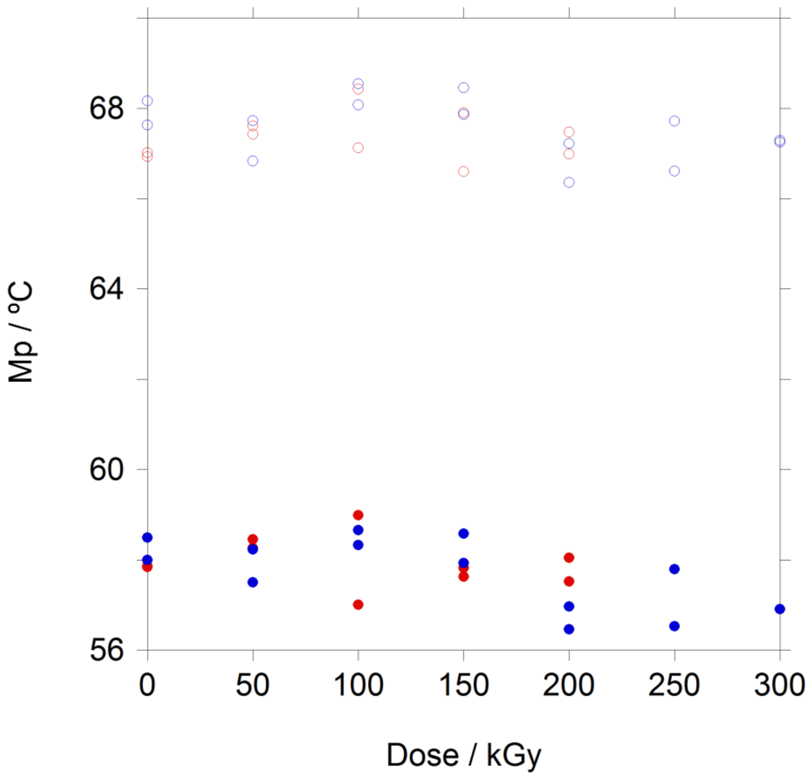

Figure S16. Melting point (M_P) for PCL 50 K (in red) and for PCL 80 K (in blue) in the first heating run (open symbols) and in the second heating run (filled symbols).

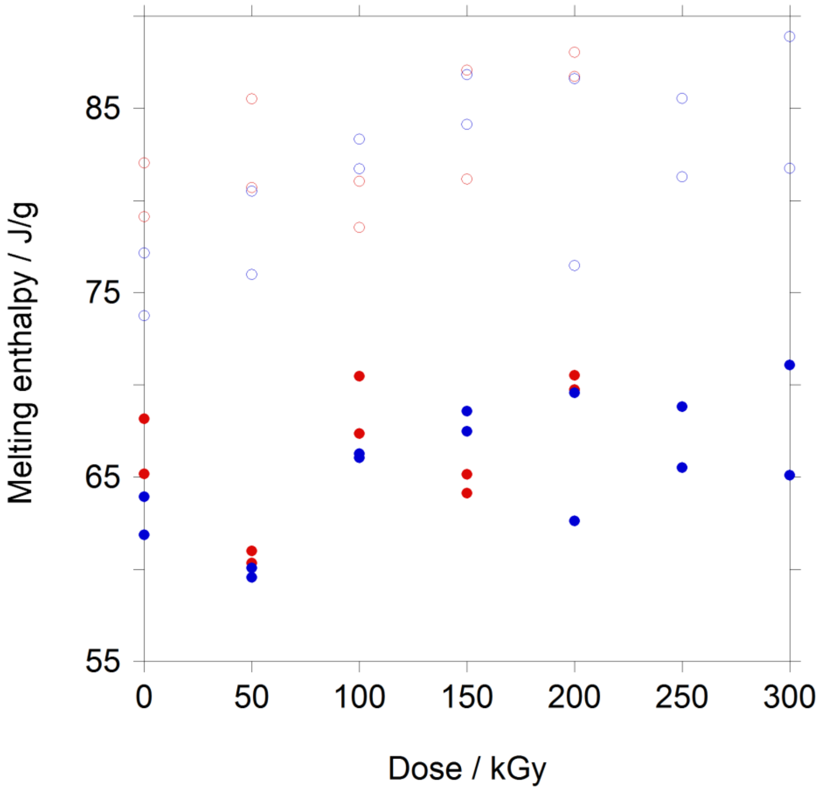

Figure S17. Melting enthalpy for PCL 50 K (in red) and for PCL 80 K (in blue) in the first heating run (open symbols) and in the second heating run (filled symbols).

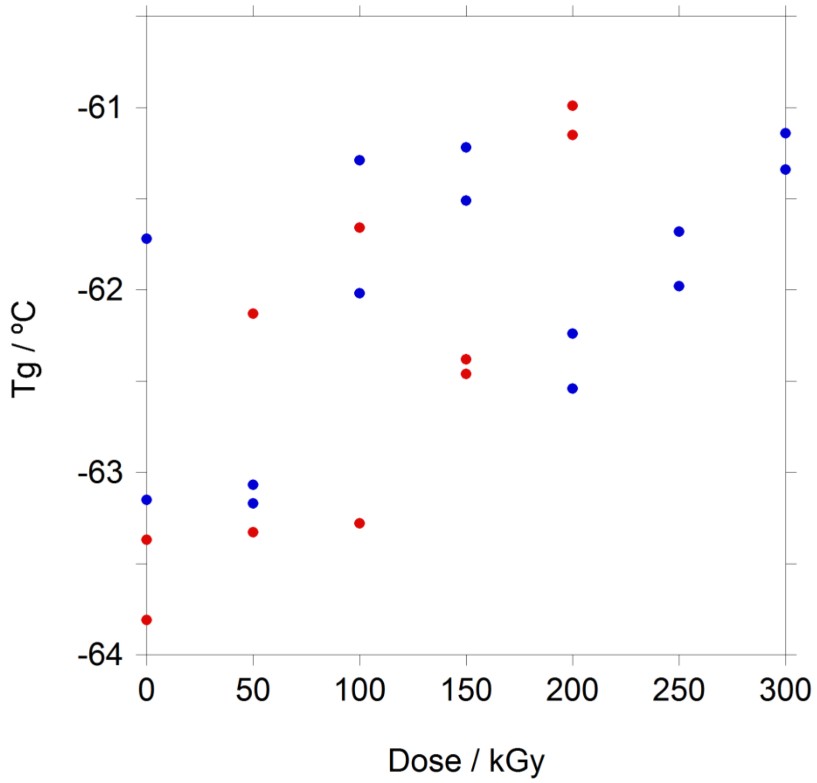

Figure S18. Glass transition temperature (T_g) for PCL 50 K (in red) and for PCL 80 K (in blue).

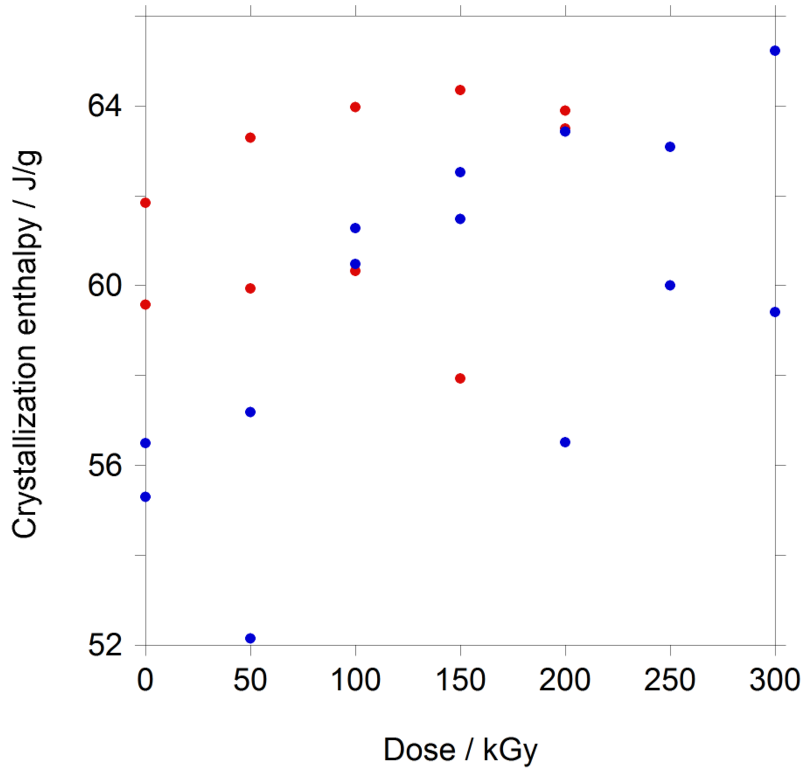

Figure S19. Crystallization enthalpy for PCL 50 K (in red) and for PCL 80 K (in blue).

Table S1. Values for tensile stress at break (T_s) and strain at break (ε_b) of PCLs when irradiated with electron beam at several doses.

	PCL 32 K		PCL 43 K		PCL 50 K		PCL 80 K	
**Dose (kGy)**	T_s **(MPa)**	ε_b **(%)**	T_s **(MPa)**	ε_b **(%)**	T_s **(MPa)**	ε_b **(%)**	T_s **(MPa)**	ε_b **(%)**
0	23.7 ± 1.8	1010 ± 100	39.0 ± 1.0	1590 ± 60	48.4 ± 0.9	2250 ± 80	48.0*± 1.5	1900*± 70
10	18.3 ± 0.4	44 ± 13	33.5 ± 0.7	1360 ± 30				
25	18.5 ± 0.5	29.5 ± 1.1	30.4 ± 1.1	1250 ± 60				
50	18.3 ± 1.1	20 ± 3	20.4 ± 1.1	800 ± 60	37 ± 2	1410 ± 90	46 ± 2	1660 ± 100
100			18.9 ± 0.7	46 ± 17	27 ± 2	1090 ± 70	43 ± 2	1460 ± 100
150					18.6 ± 0.5	610 ± 40	34 ± 3	1200 ± 80
200					18.6 ± 0.2	44 ± 12	17.9 ± 0.5	600 ± 50
250					18.3 ± 0.1	21.4 ± 1.9	17.4 ± 0.2	300 ± 200

* Samples escaped from the grips.

Table S2. Values for tensile stress at break (T_s) and strain at break (ε_b) of PCLs when irradiated with gamma rays at several doses.

	PCL 32 K		PCL 43 K		PCL 50 K		PCL 80 K	
**Dose (kGy)**	T_s **(MPa)**	ε_b **(%)**	T_s **(MPa)**	ε_b **(%)**	T_s **(MPa)**	ε_b **(%)**	T_s **(MPa)**	ε_b **(%)**
**0**	23.7 ± 1.8	1010 ± 100	39.0 ± 1.0	1590 ± 60	48.4 ± 0.9	2250 ± 80	48.0*± 1.5	1900*± 70
**25**	18.3 ± 0.4	48 ± 15	30 ± 2	1240 ± 90	42 ± 3	1580 ± 130	47*± 2	2070*± 100
**50**	17.2 ± 0.2	13.5 ± 0.6	18.7 ± 0.4	630 ± 70	32 ± 2	1200 ± 60	48 ± 4	2100 ± 200
**75**	16.1 ± 0.9	10.2 ± 0.8	18.5 ± 0.3	50 ± 20	24 ± 3	960 ± 130	44.8 ± 1.9	1630 ± 140
**100**	13.5 ± 0.9	7.6 ± 0.7	18.3 ± 0.7	24 ± 4	22.8 ± 0.7	950 ± 30	38 ± 5	1400 ± 200
**150**					16.7 ± 0.2	21 ± 3	21.3 ± 1.4	850 ± 80
**200**	10.6 ± 0.6	5.7 ± 0.4	14.9 ± 0.5	10.8 ± 0.4	16.4 ± 0.2	17 ± 3	16.8 ± 0.2	70 ± 40

* Samples escaped from the grips.

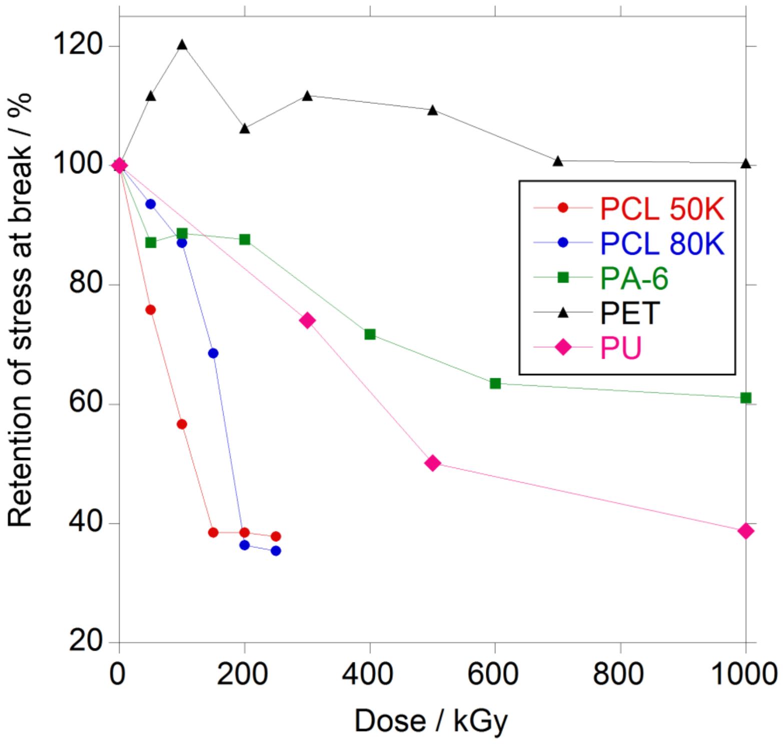

Figure S20. Retention of the stress at break vs dose for PCL 50 K (red circles), PCL 80 K (blue circles), PA-6 (green squares), PET (black triangles) and for an aliphatic polyurethane (magenta diamonds).

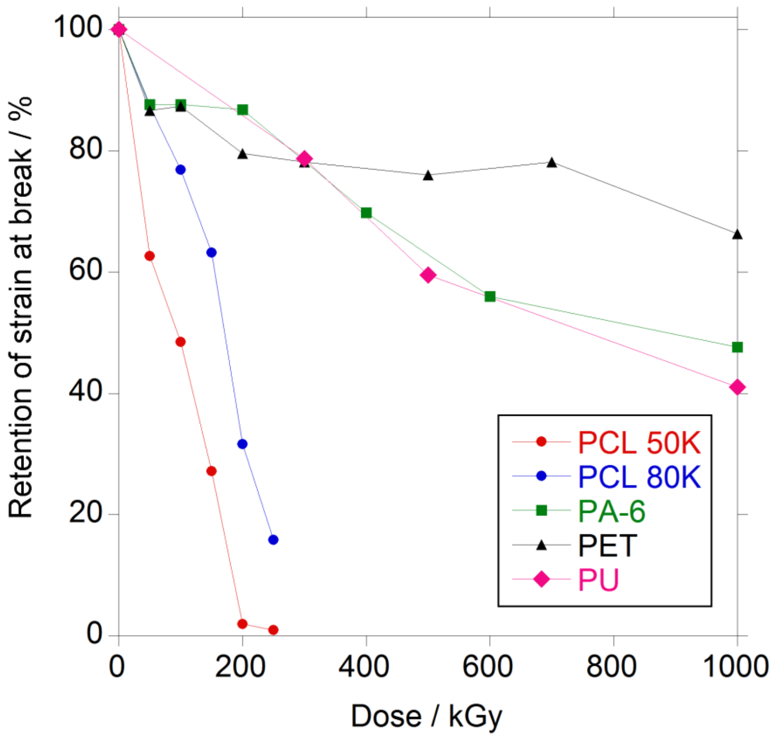

Figure S21. Retention of the strain at break vs dose for PCL 50 K (red circles), PCL 80 K (blue circles), PA-6 (green squares), PET (black triangles) and for an aliphatic polyurethane (magenta diamonds).

© 2018 by the authors. Submitted for possible open access publication under the terms and conditions of the Creative Commons Attribution (CC BY) license (http://creativecommons.org/licenses/by/4.0/).
